# A Survey on Unmanned Surface Vehicles for Disaster Robotics: Main Challenges and Directions

**DOI:** 10.3390/s19030702

**Published:** 2019-02-08

**Authors:** Vitor A. M. Jorge, Roger Granada, Renan G. Maidana, Darlan A. Jurak, Guilherme Heck, Alvaro P. F. Negreiros, Davi H. dos Santos, Luiz M. G. Gonçalves, Alexandre M. Amory

**Affiliations:** 1 School of Technology, Pontíficia Universidade Católica do Rio Grande do Sul, Porto Alegre, RS 90619-900, Brazil; renan.maidana@acad.pucrs.br (R.G.M.); darlan.jurak@acad.pucrs.br (D.A.J.); guilherme.heck@acad.pucrs.br (G.H.); 2 Department of Computer Engineering and Automation, Universidade Federal do Rio Grande do Norte, Natal, RN 59078-970, Brazil; alvarodenegreiros@gmail.com (A.P.F.N.); davihenriqueds@dca.ufrn.br (D.H.d.S.); lmarcos@dca.ufrn.br (L.M.G.G.)

**Keywords:** survey, disaster management, unmanned surface vehicle, USV, unmanned surface craft, USC, autonomous surface craft, ASC, autonomous boat, disaster robotics, floods, landslides, hurricanes, tsunamis, hazard, search and rescue

## Abstract

Disaster robotics has become a research area in its own right, with several reported cases of successful robot deployment in actual disaster scenarios. Most of these disaster deployments use aerial, ground, or underwater robotic platforms. However, the research involving autonomous boats or Unmanned Surface Vehicles (USVs) for Disaster Management (DM) is currently spread across several publications, with varying degrees of depth, and focusing on more than one unmanned vehicle—usually under the umbrella of Unmanned Marine Vessels (UMV). Therefore, the current importance of USVs for the DM process in its different phases is not clear. This paper presents the first comprehensive survey about the applications and roles of USVs for DM, as far as we know. This work demonstrates that there are few current deployments in disaster scenarios, with most of the research in the area focusing on the technological aspects of USV hardware and software, such as Guidance Navigation and Control, and not focusing on their actual importance for DM. Finally, to guide future research, this paper also summarizes our own contributions, the lessons learned, guidelines, and research gaps.

## 1. Introduction

*Natural disasters* have severe consequences for the environment, human lives, and man-made constructions. Moreover, cities and often countries face severe social and economic distress as a result of a disaster. Extreme natural events such as the earthquakes/tsunamis that occurred in Tohoku (Japan, 2011), in the Indian Ocean (2004) and the hurricane in New Orleans flood (United States, 2005) are some examples. These natural phenomena result in problems which can be felt even years after the disaster. They usually cause damage to utility infrastructure, affecting electricity, natural gas, water, sewage, communications, roads, bridges, and transportation services. Furthermore, damages at facilities such as natural gas pipes, dams, or nuclear power plants can cause even more massive disasters.

On the other hand, *man-made disasters* can produce consequences as severe as the natural ones. Oil spills, mine waste floods, heavy metal, and radioactive contamination, wildfire caused by humans directly are examples of disasters with non-natural starts. Examples are the Deepwater Horizon oil spill (United States, 2010), Chernobyl disaster (Ukraine, 1986), Bento Rodrigues Dam disaster (Brazil, 2015) and California wildfires (United States, 2018). Despite the origin of the disaster (i.e., natural or man-made), it can affect the quality of potable water, crops, and cattle, affecting food provisioning for entire regions. It can also cause catastrophic damage to nature, profoundly affecting local communities. All of those can lead to substantial economic losses, the spread of diseases, or even mental issues caused by the disaster trauma. The lack of such basic needs may also result in public social calamity.

Over the years, there has been a growing awareness [1] about disasters, either natural or man-made, and the need for measures to reduce their impact. The problem is not only the disaster itself but also how the affected region is prepared to face it [2]. Disaster Management (DM) can be divided into four different stages [3]: mitigation, preparedness, response, and recovery. The stages preceding the disaster occurrence, i.e., mitigation and preparedness, define how well the community can respond and recover from it.

The DM process currently demands better tools (more reliable, easy to use, more efficient, cheaper, etc.) for the phases preceding and after disasters. Robotics is becoming a recurrent source of tools in disaster applications. For example, regarding data acquisition applications, unmanned vehicles can play an essential role for disaster research [4] by replacing response teams in remote and hazardous environments [5], or performing long-term monitoring [6]. Unmanned vehicles have been deployed in the three main types of environments: aerial, ground, and aquatic. Most of the papers related to robotics and DM are related to ground, underwater, and unmanned aerial vehicles. Surprisingly, on this topic, the presence of Unmanned Surface Vehicles (USVs) [7] seems to have fallen behind those of other platforms [8,9], such as Unmanned Aerial Vehicles [10] (UAVs) and Unmanned Underwater Vehicles [11,12] (UUVs). As far as we known, this is the first survey of USVs for DM applications.

On the water surface, USVs stand out from other unmanned aquatic platforms for data collection due to: access to Global Positioning System (GPS) and other localization strategies [13,14]; superior communication capabilities when compared to other marine vehicles [15,16]; payload capacity; and capacity to use energy harvesting (solar, waves, wind) for long-term missions. Also, USVs can be seen as moving sensors, which can quickly perform several measurements in different locations, or even be deployed for emergency relief tasks in remote areas.

Hence, the *main contribution* of this paper is a systematic review of USVs for DM and their role in disaster scenarios. Even though this work aims to be complete, for example, by addressing either natural or man-made disasters, it excludes disasters caused due to homeland security breaches (e.g., bombs) and standard water-life- or water-monitoring tasks. Finally, we present general guidelines for USVs focusing on DM.

This paper is presented as follows. Section 2 describes the basic concepts in DMs, key influential papers related to disaster robotics, and existing surveys about USV that not related to disaster applications. Section 3 presents papers where USVs were deployed on natural disasters such as earthquakes, tsunamis, hurricanes, floods, and landslides. Section 4 surveys USV papers related to chemical, biological, and radioactive hazards. Section 5 presents structural inspection and search and rescue (SAR) applications related to disasters. Section 6 discusses the related work including guidelines for USVs deployment for disaster. Section 7 describes our work in the context of USV research for disaster applications. Finally, Section 8 presents our concluding remarks.

## 2. Groundwork

This section describes basic concepts related to the DM cycle, the role of robotics in this cycle, and existing survey papers related to USV.

### 2.1. Disaster Management

**Figure 1:**
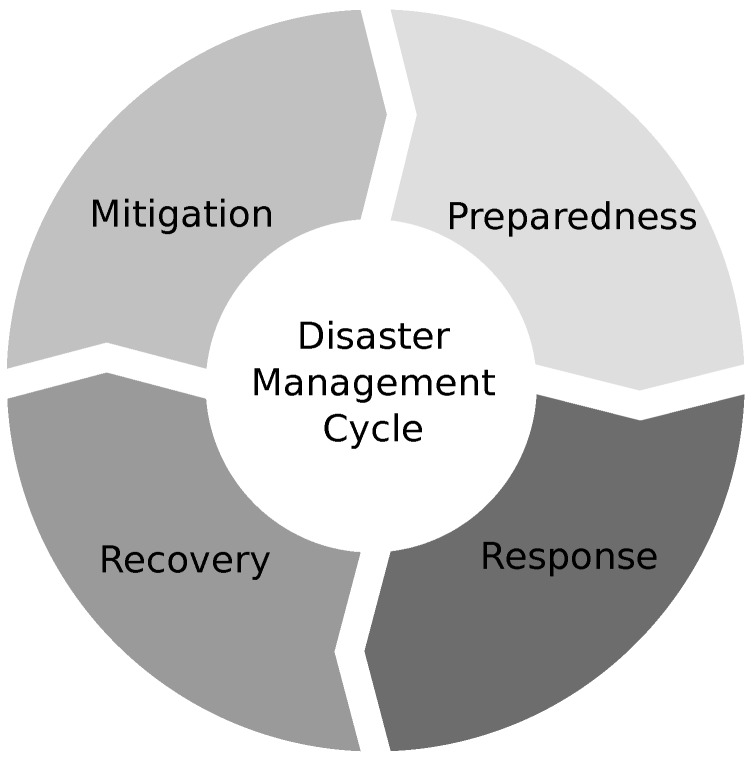
The DM cycle. Mitigation and Preparedness occur before the disaster indeed, while Response is immediately before and after it. Recovery always happens after the occurrence of a disaster. Note that the DM cycle tends to make affected regions better prepared when another disaster strikes.

DM involves different actions which depend on previous preparation and response capabilities when a disaster strikes. Such actions often occur in harsh conditions (in emergency scenarios) or in preparation stages where conditions are more favorable. As such, DM is usually divided into four (Figure 1) different stages, occurring after and before a disaster strikes [3].

- **Mitigation**: Actions before disaster strikes. It consists of all types of actions taken to identify vulnerabilities and reduce or eliminate the risks of future events, such as the permanent removal of affected populations from dangerous areas and reinforcing weakened structures prone to collapse.
- **Preparedness**: Actions before disaster strikes, strongly tied to the mitigation process. However, preparedness works with the assumption that hazards cannot be avoided entirely. The goal is to devise workarounds and preparations when the disaster occurs, such as the creation of evacuation plans, training & warning systems [17].
- **Response**: Immediately before, during, and after a disaster occurs. It consists but it is not limited to evacuation, rescue, and needs assessment to save lives and minimize the damages on properties.
- **Recovery**: Actions which take place after a disaster. They aim to reestablish life as it was before the disaster. It involves the reconstruction and monitoring of affected communities/areas.

When it comes to in situ tasks involving DM, there are different options, including rescue team incursions by land, air, or water. Still, affected regions can be dangerous for the rescue team—e.g., 9/11 terrorist attack or the Fukushima Daiichi nuclear accident, where first-responders heroically gave their lives to save others. These situations are examples where disaster robots can play a crucial role, keeping the rescue team safe or even freeing them to execute other tasks.

### 2.2. Groundwork on Disaster Robotics

Even though there are currently plenty of work focusing on robotics and DM [18], the presence of USVs [7,19] directly associated with DM seems to have fallen behind those of other platforms [8,9], such as UAVs [10] and UUVs [11,12]. For instance, Wong et al. [20] address robotics and automation systems for harsh environments, but briefly discusses USVs with a generalist focus. The reference book from Murphy [18] is an important reference for disaster robotics, covering different unmanned systems, including USVs and UUVs where they were actually used. In fact, Murphy uses the umbrella term Unmanned Marine Vehicles (UMVs) for such group of robots. There, UUVs seem to be predominant when compared with the use of USVs. Cubber et al. [21] present a book focusing mainly on marine vessels for SAR, where the authors point out the low prevalence of research involving marine vehicles for SAR, with a strong focus on the authors projects.

The main objective of the present work is to map the actual contributions of USVs for DM.

### 2.3. Existing Surveys about USVs

The research starts with a critical review of key surveys related to USVs and also DM [22,23,24,25,26,27,28,29,30,31,32,33,34,35,36,37,38,39,40,41], most focusing only on USVs, where a few vaguely focus on USVs for DM.

Rodriguez et al. [30] address research on environmental monitoring for USV. Their focus mostly on oceanic survey applications such as mapping/cleaning oil spills, weather/storm forecasting, and water sampling. The survey also lists challenges for real-world applications such as the system endurance for long-term missions and operation under extreme weather conditions. Obstacle avoidance (both above and underwater) is also pointed as a technological challenge. The lack of laws and regulations for the use of USV is a non-technological challenge which increases the investment risk, reducing the number of business opportunities and technology evolution. The authors included the results from interviews with specialists from different areas who are potential users or clients of the USV technology. Bayat et al. [42] studies search techniques for environmental monitoring where USVs are marginally mentioned. Marques et al. [43] studies marsupial robotic teams for environment monitoring of water bodies. The authors claim marsupial robotic teams are especially tailored for water body monitoring, due to their versatility, where each robot can compensate the limitation of others. A survey on robots for environmental monitoring is presented by Dunbabin et al. [44] presents a span of robot applications for environmental monitoring where some USVs mentioned in the present work are mentioned—e.g., the Wave Glider USV. Overall, the current environment monitoring surveys do not seem to address USVs for DM usage in a specific way. Lattanzi and Miller [45] review structure inspection using robots. There, a couple of works using USVs are mentioned, one using USVs in post-disaster cases. They conclude that current work on structure inspection often involves case specific needs for sensors and robot motion in challenging environments, with a trend toward non-destructive analysis. The two significant challenges for structure inspection using robots involve massive robot data manipulation and the need for increased autonomy for such robots. A more recent survey on unmanned systems for construction applications is described in Moud et al. [41]. The authors highlight the relevant presence of USVs for structural inspection after disasters. Key DM works on structural inspection, in the scope of the present work, are mentioned.

Schiaretti et al. present two sister papers, divided into parts A [37] & B [38] that focus on a clear classification of the levels of autonomy for USVs. Part A presents an approach which divides the autonomy problem of USVs into subsystems and corresponding scores: decision-making (1–10); action taking (1–10); exceptions handling (1–10); and cooperation subsystems. The authors introduce the cooperation subsystem as new information in comparison to the related work presented in the paper. Moreover, the authors argue that developed exception handling and cooperation subsystems are evidence of higher autonomy levels, stating that autonomous Guidance Navigation and Control (GNC) is common to almost all USVs. The authors offer a table with possible combinations of overall subsystem scores and global scores. However, the entire underlying rationale to the scoring system is not explicitly presented. Part B of the same autonomy survey classifies 60 USVs, developed over more than 20 years, according to the autonomy levels defined in part A. They evaluate the USVs and show that most are only able to perform autonomous path following—i.e., Level 3. They also present data showing that most prototypes correspond to scaled models, where the most common type of hulls used are single and double hull (catamaran), while the engine is typically electric with batteries—sometimes charged with solar panels. The most advanced systems rely on obstacle detection and avoidance using cameras or Light Detection And Ranging (LiDAR). They conclude that the level of autonomy of USVs is rising and that research on USV cooperation is increasing. Thompson and Fletcher [46] discuss mission planning for AMVs, covering some of the work on DM involving USVs related to SAR and structure inspection. The following end-user requirements for mission planning are highlighted: survivability; reliability; quality of mission outcomes; and utility.

There is plenty of work related to USVs focused on control, most likely due to the harsh conditions which may happen due to waves, currents and winds a vessel may face [39]. The groundwork of Fossen [22] surveys non-linear control of ships. Ashrafiuon et al. [26] present a review of non-linear tracking and setpoint control for USVs. Qi et al. [32] present a survey on motion control for USVs and UUVs considering different types of propellers. Azzeri et al. [33] review course keeping control systems for USVs. Xiang et al. [47] reviews the use fuzzy logic for the control of USVs and UUVs. Kumru et al. [36] present a brief survey focusing on tactical control algorithms for USV path tracking. Liu et al. [35] give a detailed review on USVs, focusing on GNC, but also addressing design & typical sensor characteristics, comparisons with other platforms, such as satellites and UUVs, hybrid cooperation between USVs and other unmanned systems, as well as current applications in a broad sense. Shi et al. [39] describe a review of marine mechatronics focusing on USVs, UUVs, and other marine devices. Regarding USVs, the paper presents three main challenges: the non-linearity of the system; velocity measurement errors; and localization noise—probably the latter is a consequence of the former two. The article performs an overview of techniques involving Dynamic Positioning (DP) control, path tracking, trajectory tracking, and modeling of system uncertainties. Campbell et al. [29] focus on the review of existing USV guidance and motion planning methodologies which could be used to implement naval obstacle avoidance rules based on the International Regulations for Preventing Collisions at Sea (COLlision REGulations at Sea—COLREGS). According to the authors, the current research problems for USV obstacle avoidance include the ability to work in the presence of environmental disturbances (e.g., waves, currents, wind, etc.) and to operate in real time. Problems also include research involving multiple USVs in formation. More recently, Liu et al. [40] perform a survey on formation control focusing on unmanned vehicles, also including USVs.

USV designs and prototypes are surveyed by Caccia [23] and later, very briefly, by Othman [31]. Manley [25] reviews 15 years of USV development, mentioning some reference works. The author reinforces the need for research involving USVs and the COLREGS, without direct references to USVs for disasters. Bertram [24] surveys USVs with focus on applications. Prototypes, which are mostly military, for SAR missions are briefly mentioned—i.e., the Rescue Dolphin (1995), Seal, and the Search and Rescue Portable, Air-Launchable (SARPAL) USVs. Motwani [28] presents applications and an overview of different USVs where SAR USVs are also mentioned—e.g., the Sterling USV. The survey reveals a tendency, in the last decade, to design small-sized USVs. This trend is seen as a critical change in USV research, mostly restricted to the military domain at the time, which eventually enabled USV research in the civil domain as we see today. Zereik et al. [48] discuss the numerous projects involving marine robotics, where some DM-related projects are summarized.

Regarding DM-related works, Murphy [49] briefly reviews a decade of rescue robots, up to 2012, when the presence of USVs for DM was small. Similarly, Bogue [19] analyzes the viability of SAR robots, but the use of USVs are briefly discussed. Maurer et al. [7] review works on Urban SAR, also referring to USVs only marginally. In a recent survey on multihop networks for aerial and aquatic robots, Sanches-Garcia et al. [50] conclude that most works related to the topic focus on general applications, while disaster specific works lead those involving specific applications. In harsh post-disaster scenarios where the communication infrastructure (e.g., antennas, base stations, etc.) has been destroyed, IEEE 802.11 standards for ad-hoc networks and satellite technologies are eligible for use. However, the restrictions imposed by the latter—e.g., permissions, charges for use, among others—make IEEE 802.11 a more feasible solution to establish communication links between first-responders. We have identified a stronger focus on UAVs than USVs in the survey. However, the authors claim the networking strategies used for UAVs could be extended to USVs—except for lower layers where sonar, for instance, may play a role. The books from Murphy [18] and Cubber et al. [21] are key references for our work. Still, from the works above, only [21] focuses mainly on marine vessels for DM, but with a strong focus on the authors’ projects. Murphy et al. [51] presents a chapter with excellent references and guidelines for SAR Robotics, where challenges involving training and testing are raised. The chapter includes some relevant work on USVs mentioned in this paper. Please note that key works on DM always treat USVs together with other robots.

**Figure 2:**
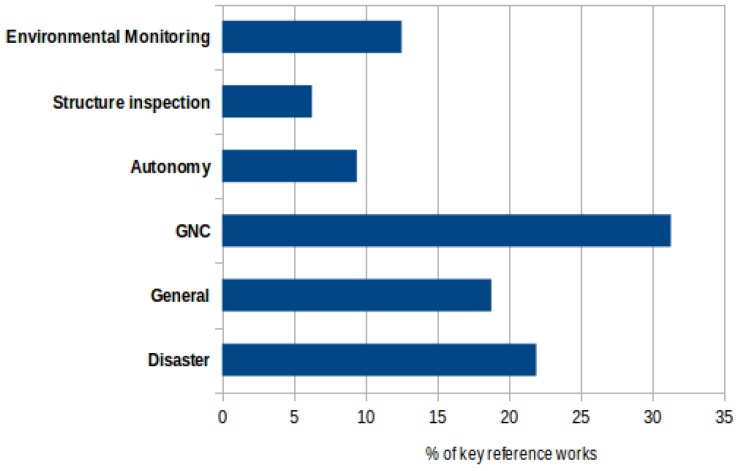
The distribution of surveys analyzed. From those associated with DM, only one is dedicated to UMVs, but none to USVs exclusively. Please note that if we remove works exclusively focusing on USVs from the list, the number of surveys drops to zero.

Figure 2 summarizes the current surveys involving unmanned systems and DM. There are no surveys dedicated to USVs where the focus is on DM, demonstrating the *novelty* of this paper. This chart is organized in six categories:

- Disaster management [7,18,19,21,49,50,51]
- General surveys regarding USVs and unmanned systems [23,24,25,28,31,48]
- Guidance, Navigation and Control [22,26,29,32,33,35,36,39,40,47]
- Higher-level autonomy [37,38,46]
- Structure inspection [41,45]
- Environmental monitoring [30,42,43,44]

By and large, the present work highlights the massive presence of papers focusing on some kind of control over the USV or groups of USVs. Some works focus on general applications, while the few brief surveys related to DM do not have a strong focus on USVs. The presence of USVs for DM deserves an exclusive survey to isolate its importance in the hall of unmanned systems.

### 2.4. Survey Protocol

In this paper, the research focus is the direct use of USVs in natural and environmental disasters. Homeland security problems, environmental monitoring tasks, and general-purpose works—e.g., water monitoring, control—are beyond the scope of this work. This work focuses on peer-reviewed papers, both conference and journal papers. Therefore, unless we deem necessary, we avoid referencing technical reports or book chapters.

The research protocol involves an initial search using conventional research databases such as Google Scholar and IEEE Explore, using the keywords from Table 1.

Then, we performed a snowball search approach [52] to cover references and citing work. Each work was included or excluded according to a top-down analysis of the title, abstract, and text body. General-purpose works that only mention disasters as motivation are out of scope since our focus involves USV research directly associated with disasters.

Two major challenges of the research protocol are related to the restriction of the number of characters of search engines and the large and different expressions used as alternative nomenclature for USVs. Our approach to solving these issues was to use regular expressions and also separate the search by disaster type or application as the research evolved. Another challenge for this work is the proximity of terms involving unmanned systems and disasters, which we could not avoid. For example, the term “flood” is also used in computer networking, which means sending a packet to every outgoing link. Concerning nomenclature, we decided to use only the term USV in the whole paper, despite possible differences from remotely operated and autonomous surface vehicles. The final challenge of the present endeavor was the fact that it involves a multidisciplinary field, where works are spread across many publications. As a workaround, we have split the problem into parts where team members addressed specific subareas, namely SAR, structure inspection, specific disasters associated with USVs, and contaminants— i.e., radiation, chemical, and biological ones.

From thousands of works, we narrowed the relevant papers which are presented in the remainder of this paper.

## 3. USVs for Natural Disasters

This section presents the current USV research focused on specific natural disasters, namely: Tsunamis & Earthquakes (T. & E., Section 3.1); Hurricanes (Section 3.2); Floods (Section 3.3); Landslides & Erosion Risks (L. & E., Section 3.4). Table 2 summarizes the surveyed papers that deal with the application of USVs to natural disasters, where *NR* means that the authors did not report the field in the corresponding paper.

### 3.1. Earthquakes & Tsunamis

Earthquakes are sudden and shivering tremors that shake the surface of the Earth, with varying intensity and duration. The most common cause of earthquakes is the underground shock of tectonic plates. When earthquakes happen offshore, depending on their intensity, they can cause tsunamis. Characterized by the displacement of large bodies of water, tsunamis can be generated whenever the seafloor undergoes sudden deformations and create vertical displacement of the body of water. Tsunamis and earthquakes are extreme events that can cause vast destruction and damage miles away from their focus. Therefore, early detection is essential for evacuation actions and to reduce the number of fatalities. Tsunami and offshore earthquakes can be detected by monitoring seafloor conditions such as pressure created by crustal friction and off-beat water surface height.

For tsunamis and earthquakes detection, studies present the usage of USVs with GPS/Acoustic (GPS/A) devices, seafloor pressure sensors, seismometers, and hydrophones. Takahashi et al. [53,54] use a USV as a central base of a system for detection of crustal deformation. The system is composed of a GPS/A transducer attached to the USV in the sea surface, a seafloor pressure sensor, wired to the USV, and six GPS/A transponders in the seafloor. Kido et al. [55] present the progress of a four-year project of the Tohoku University. Together with Nagoya University and the Japan Coast Guard, they use a USV as a survey station for crustal deformation detection. The USV is equipped with a GPS/A device for collection of seafloor acoustic transponder measurements around the Japanese Islands.

Sukhovich et al. [56] use a USV (named MERMAID) equipped with a hydrophone for seismic monitoring. The hydrophone detects acoustic signals generated by seismic waves from Earth’s interior. The MERMAID USV was launched near the Indian Ocean triple junction on the 24th of November 2013 and detected an earthquake swarm that was followed by the main event of magnitude 5.1.

Berger et al. [57,58] use a Wave Glider USV as an intermediate station for data transmission between a seismometer on the seafloor and a land station. Carragher et al. [59] discuss the usage of Wave Glider as USV for retransmission of seismic data collected from the seafloor DART (Deep-ocean Assessment and Reporting of Tsunami) network through GPS/A. The DART network is maintained by the US National Oceanic and Atmospheric Administration (NOAA). It has 39 DART monitoring stations in its network. Each DART station consists of a seafloor bottom pressure recorder (BPR) with a surface buoy anchored next to it. An acoustic link transmits data and commands between the buoy and the BPR, which collects pressure readings.

Another way to detect tsunamis is through estimation and comparison of sea-surface height. Maqueda et al. [60] present a Wave Glider equipped with a geodetic GPS and uses Precise Point Positioning (PPP) for determination of water surface heights in Loch Ness, with a precision of around 0.05 m. PPP uses real-time satellite signals to derive orbital and clock and can be a tool to measure tsunami wave height [54].

### 3.2. Hurricanes

Hurricanes or typhoons depending on the part of the Earth are tropical cyclones with wind speed higher than 74 mph (about 119 Km/h) that are created due several factors such as the sea-surface temperature, low tropospheric moisture, sea-level pressure etc. [78]. Hurricanes can have disastrous effects, including casualties and damage to buildings. Their effect on the sea can even lead to floods which can cause further problems to the affected region. Such disasters can be monitored from space, as well as by the forecasting of ocean behavior as they pass. In this sense, USVs can serve as a tool in the event of hurricanes, for weather forecasting, disaster response, and recovery missions, providing observations that are not presently available through manned platforms and satellites. In such extreme events, USVs may be used since it avoids the high risk to personnel operating in these dangerous and remote environments [65]. Below are described the current applications of USV for disasters caused by hurricanes.

For the recovery phase of DM, Murphy et al. [61] investigate the cooperation of a USV with a rotary-wing UAV to detect damages to seawalls and piers. The USV named AEOS-1 is a catamaran design with two polyethylene pontoons connected with a central T-shaped chassis that supports the instrumentation. The USV is designed to determine the extent and severity of the damage to the seawall and bridges. It is equipped with a camera to perform the inspection above the waterline, while an underwater acoustic camera detects structural damage below it. The UAV is a battery powered T-Rex miniature helicopter, which contains a miniature pan-and-tilt visible light camera to provide a bird’s-eye view. This view allows the USV pilot to localize the vehicle for navigation relative to the structure, as well as identifying when GPS data is not available. Tests were performed in Marco Island after Hurricane Wilma (2005) to check the USV mobility to work underneath small docks, as well as how it operates around bridges in significant current. It was observed that the USV offered advantages over manned surface vehicles and UUVs since it is easier to deploy in disaster conditions, reaching places manned boats cannot reach. Furthermore, the bird’s-eye view provided by the UAV can help with general safety and control by determining safe lanes for sea navigation. The cooperation of the USV with UAV is further applied in inspecting littoral environments for military and environmental applications [79].

For post-disaster bridge inspection after hurricane Ike, a category four storm that struck Galveston, Texas in 2008, Steimle et al. [62] and Murphy et al. [63,64] use a combination of a Sea-RAI USV with two UUVs (YSI EcoMapper and a tethered VideoRay) to inspect the bridge footings by searching for scour and mapping the debris field around the bridge. The Sea-RAI USV is a platform based on two 6ft catamaran hulls that carry an acoustic camera for sub-surface inspection and three video cameras for viewing above the waterline. Tests to assess the bridge substructure were performed in the Rollover Pass Bridge located in Galveston Bay, Texas. They found out that it is essential to map the debris first with USVs, and then use the mapping as input for UUV collision avoidance. GPS problems near the bridge piers were also reported during the inspection of affected structures.

Lenain and Melville [67] perform observations of the ocean’s response to a tropical cyclone (hurricane) using a Wave Glider [80,81]. The glider left from San Francisco, California, heading to Australia, came close to the category three Tropical Cyclone Freda (2012). The closest approximation of the glider with the tropical cyclone took place near New Caledonia, where the glider collected data of the evolution of the wind, the directional wave field, the sea-surface temperature, and the Stokes drift profile as Freda passed near the vehicle. Measures obtained by the glider agree with recent hurricane marine boundary layer studies [82]. With the success of such measurements, the authors conclude that the glider allows an extensive use of this technology in measuring air-sea interaction processes in extreme conditions.

Fitzpatrick et al. [68] describe the results of a 100-days journey of a Wave Glider platform in the Gulf of Mexico. During this period, the glider collected surface weather, water temperature, wave, and ocean current profile data within tropical cyclones. It collected data from the tropical storm Hanna in the Caribbean Sea to validate the data against that from nearby buoys. Numerical models were created to predict tropical cyclones and their intensity. Also, collected data was used to understand the Wave Glider maneuverability capabilities in different wave and current conditions. Results indicate an agreement between data measured with the USV and data from the nearby buoys in fair conditions and on the periphery of the tropical cyclone. Lessons learned suggest that tampering or possible collisions can be addressed by using more visible signage on the glider, by deploying the USV in minimally trafficked regions, and by increasing the distance from buoys during loitering exercises.

Mitarai and McWilliams [69] also use a Wave Glider to monitor surface winds and currents to understand oceanic responses to tropical cyclones. Such monitoring gives a more comprehensive view of actual atmosphere-ocean interactions in a typhoon, as well helps to accurately model air-sea coupled processes. The authors affirm that the Wave Glider was chosen since the onboard weather station is designed to work properly on a moving platform, under severe sea conditions. Such gliders demonstrated an ability to weather more than 10-foot seas and more than 40 kt winds [80], surviving five hurricanes and three tropical cyclones and traveling more than 560,000 km (300,000 nautical miles) [59]. The USV is equipped with an acoustic Doppler current profiler (ADCP) and a conductivity-temperature-depth sensor, while the weather station collects air temperature, wind speed and direction, and barometric pressure. Observations using the glider were conducted on the ocean surface 150 km east of Okinawa, Japan during the Typhoon Danas (2013), which is equivalent to a category 4-hurricane. The glider entered the typhoon eye area collecting a time series of surface winds and currents in typhoon cores to examine the balance between wind-induced energy and the increased kinetic energy of the upper ocean.

Patterson et al. [65,66] present the development and testing of a monohull USV called EMILY for exploration of bathymetry, littoral mapping, and tracking hurricanes. EMILY can operate autonomously and is equipped with meteorological sensors for measuring atmospheric conditions and water temperature in the ocean. Collected data can be stored on board or be transmitted via radio or satellite communication links. According to the authors, the data collected by the USV can be used for extending hurricane landfall times, improving storm forecast accuracy, and providing information to emergency managers and the public. Simulations are performed to test ways to enhance the possibility of the USV approach a hurricane.

Considering the application of robotics to disasters in general, Murphy et al. [83] summarize the activities and lessons learned from a set of four responses (La Conchita Mudslides, Hurricane Dennis, Hurricane Katrina, and Hurricane Wilma). Lessons learned include the low performance of vision systems under low temperatures, the requirement of careful placement of cameras to avoid collisions, the recording of Human-Robot Interface data for future analysis, and the need for more realistic simulations for training purposes.

### 3.3. Floods

Floods are natural events caused by an overflowing of a large amount of water that submerges or inundates a piece of land that is usually dry. Floods are common after-effects of different extreme events, such as hurricanes, dam ruptures, massive storms, and tsunamis. They affect several million people each year, being one the most significant natural hazards our society is currently subject to [84]. In fact, the total population located in zones prone to flooding have increased dramatically over recent decades and are expected to increase further [85]. As indicated by Jongman et al. [85], the most affected people inhabit underdeveloped countries, which lack early warning systems, flood control, and emergency response infrastructure. Scerri et al. [70] affirm that USVs are ideal to effectively address this problem since they are simple, robust, and reliable, being ideal for flood mitigation and response. Current applications of USVs for disasters caused by floods are described below.

Scerri et al. [70] present a technical description of the problem from the perspective of multi-agent systems, where some cooperative boats might be deployed to provide situational awareness over a small area. In this sense, a set of autonomous boats should provide situational awareness, damage assessment, and deliver supplies before more traditional emergency response assets can access affected areas. They suggest the use of airboats [71] since they are flat-bottomed boats with an above-water fan to propel themselves forward safely and effectively through shallow or debris-filled water. Airboats may also collect water, checking for diseases carried by the flood.

Mancini et al. [72] use a combination of USV [8] and UAV to map a river/estuary since it is fundamental to aid the monitoring during critical events as heavy rains that could produce flooding. While the UAV generates ultra-high-resolution imagery from a bird’s-eye view, the USV maps the riverbanks with more details, collecting images from different points of view enhanced by an RGB + depth (RGBD) sensor (i.e., a camera that provides both color and dense depth images). Using a USV is important mainly in areas that require maintenance due to a significant presence of canopy that occludes the identification of the riverbanks. The authors choose a small USV since small river/basin require vehicles with the capability to navigate in the presence of shallow water or presence of canopy or algae. Tests are performed in the Province of Ancona, Italy, to detect short-term changes, i.e., identify the changes occurred in the river basin during a strong rainy event.

Zhang et al. [73] use a combination of a USV and a UAV in an aerial-surface system for rescue operations in flooded areas. In this system, the USV carries a UAV into the complex stricken area to acquire information and rescue survivors. While the USV navigates in the area, the UAV takes off from the USV and sends global information about the environment, such as photos and videos. When the mission is finished, the UAV should be capable of landing back on the USV automatically. In this configuration, the USV is responsible for collecting local environmental information with laser and camera to generate a map for navigation planning, and for releasing rescue equipment to survivors (e.g., a rescue rope throwing equipment to throw the rope to the survivors). The combination of USV with UAV allows the USV to obtain global information for trajectory planning while creating a local path with its local information. Xiong et al. [74] provides a complete description of this flooding disaster-oriented USV.

Li et al. [75] deal with the problem of measuring the vertical profiles of horizontal flow velocity for estimations of flushing time, fluxes of water, salt, suspended sediments, and other waterborne materials. These measures can be used to understand and predict floods in case of extreme weather events in water channels—traditional methods to measure the current fixes the instrument at a specific location to collect a long time series of flow data. Another approach runs a vessel in a transect line repeatedly over a complete tidal cycle. In the former, the instrument cannot obtain accurate quantification of the total transport, and the latter is labor intensive and weather dependent, not feasible for long-term observations. To overcome such drawbacks and perform accurate measurements of the cross-channel structure and long-term estimate of the total transport, Li et al. develop a USV equipped with an ADCP sensor to operate continuously for covering both flood and ebb during a complete tidal cycle. Tests are performed in a tidal channel at Port Fourchon, Louisiana.

### 3.4. Landslides & Erosion Risks

Landslides are phenomena caused by the slipping of solid materials, such as soils, rocks, vegetation, and building material along sloping terrain. It occurs in areas of rugged relief, from which the original vegetation cover was removed, responsible for soil consistency and for preventing the flow of water through the roots. The landslide differs from the erosive processes by the amount of mass transported at a high velocity. These natural phenomena cause immediate problems for the population, regardless of their social condition, and for the environment. In this type of disaster, USVs can conduct underwater three dimensional (3D) surveys using sonars/side scanners to find underwater landslide signs. Another associated problem involves submarine landslides, which can affect man-made structures such as the massive gas and oil extraction platforms in the ocean [86]. Moreover, an underwater (or near the coast) landslide can even cause tsunamis [87].

Accurate hydrographic surveying and coastal monitoring are essential for numerous reasons: coastal flood zone modeling; estimating storm and tidal surges; quantifying volumes of sand movement due to erosion and accretion [88]. In particular, coastal erosion and accretion (silting) is a major concern nearby populated zones [89,90], as silting can increase the impact of floods and erosion might affect the structure of nearby buildings and lead to landslides. By accurately comparing the elevations in ocean tides, emergency agencies could plan evacuation routes for coastal communities in case of events such as imminent landslides caused by the elevation of the water surface.

Ferreira et al. [76] use the ROAZ USV [91] for risk assessment in shallow water and marine coastal regions. The USV was remote-controlled and used for wave modeling, and for searching for rocky outcrops and sandy patches. The accuracy of the bathymetric survey was not informed, even though Bio et al. [77] point out that accuracy can vary about 30cm, depending on observation conditions in the water body and sensor precision.

Bio et al. [77] conduct two surveys involving the risk of erosion and landslides next to the coast. They have used a remote-controlled USV, during high tides to get as close as possible to the shore, to perform sub-tidal bathymetry with sonar imagery. They classify the erosion risk in each monitored area in three classes: low, intermediate, and high risk.

## 4. Use of USVs in Environmental Disasters with Contaminants

Maritime environmental disasters generally occur after collisions, grounding, stranding heavy weather, seismic events, explosions, or fire. What follows may be oil spillage, bunker, dirty water, or harmful chemical substances, with serious impacts on the environment and affected communities [92,93]. Our focus here is not to survey water-monitoring USVs. Instead, we focus on major problems which may involve more than standard water sampling tasks. We have chosen to divide such events into three major topics: chemical (Section 4.1); biological (Section 4.2); and radioactive (Section 4.3) hazards.

### 4.1. Chemical Hazards

Although hydrocarbons can naturally occur at sea through seepages on the ocean floor, oil & gas in large quantities can bring environmental risks to water life and man-made constructions at sea. The presence of oil spills from natural sources is traditionally used to indicate the presence of underwater oil fields, a task which can even be performed by USVs [59,94], resulting in substantial economic growth of a region. However, oil & gas spills are typical sources of environmental disasters [95,96,97], profoundly affecting water life and local economies. Environmental disasters involving oil pollution entail catastrophic consequences for marine habitat. They often spread out hundreds of nautical miles from the source of an incident and cause severe harm to the maritime environment [92], affecting birds, mammals, and mainland shorelines. Such disasters are even more destructive when harsh chemical solvents and cleaning materials are used. Studies from Samuelides et al. [98] show that the most significant maritime environmental disasters were caused by oil spillage from ship collisions or grounding at sea.

The recent history of oil spills has clearly shown their catastrophic effect on coastal ecosystems. Oil spills have been taking place at sea since the early days of offshore oil extraction and oil-carrying tankers [99]. The largest oil spill in the history of the petroleum industry occurred in 20 April 2010, with the Deepwater Horizon Macondo semi-submersible offshore drilling rig in the Gulf of Mexico: 210 million gallons of crude oil were released, affecting 180,000 km of ocean surface, where 39,000 personnel, 5000 vessels, and 110 aircraft were involved in cleaning, and over 700 km of booms were deployed [100]. In this disaster, autonomous marine robots played a pioneering role in fighting an oil spill [101]. Since then, many approaches using USVs have been studied to detect and mitigate the effects generated by such disaster, avoiding human exposure to hazardous conditions and reducing the cleanup labor costs. Table 3 presents the work that use USVs to deal with oil spill problems, where the papers are grouped by task— *NR* means “Not Reported” to data not reported by the authors.

#### 4.1.1. Oil Spill Detection and Monitoring

The detection and localization of pollutant sources, such as oil spills, is an important research topic for the mitigation of the impact of such sources on the environment by allowing an effective control strategy. Studies have shown that the impacts of oil spills can be minimized by the existence of an efficient and effective spill response plan [128], with the initial hours following an oil release as the most crucial for mitigating the extent of impact on the environment [129,130]. In this task, the aim is to find the location of a region that is the source of the substance of interest (e.g., oil spill) using a single or a group of cooperative unmanned vehicles [42], equipped with cameras or laser fluorosensors for detecting and classifying oil [131]. The latter is preferred over the former, since cameras may suffer from poor contrast and lack of positive discrimination, while laser fluorosensors use the phenomenon of ultraviolet light absorption by aromatic compounds in petroleum oils, which become electronically excited, and thus enable its operation either during the day or night [132]. UUVs and USVs are especially tailored to deal with such issues since they can carry out tasks in a variety of environments without jeopardizing human life [133].

Most works on oil spills detection use either UUVs [107,134], since they try to find the underwater location of the oil spill, or UAVs [135] since it is easier to estimate the amount of oil on the surface. Liu et al. [105] affirm that in areas where oil spills frequently occur, such as coastal ports and oil drilling platform surroundings, the use of USV is seemingly more convenient when compared to traditional airborne and shipborne laser fluorosensors. Shipborne fluorosensors are not as agile or versatile as USVs, while airborne ones are more suitable for wide area detection. Moreover, unlike USVs, airborne cannot provide the precise information about the position of the slick on a continuous basis [136].

Ferri et al. [102,103] present a compact vehicle called HydroNet USV, which was designed to detect hydrocarbon, heavy metal concentrations (chrome—Cr(VI) and Cr(II), mercury—Hg(II) and cadmium—Cd(II)), and oil slick in real time using custom-made miniaturized onboard sensors. The USV is designed for long-range missions, lodging an onboard water analysis system. It was tested during a field test spanning 12.5 km along the coast of Livorno, Italy. Later, Fornai et al. [106] adapt the HydroNet to collect and store or process onboard water samples from up to 50 m down the water column. The system was again tested in field trials in Livorno, but they do not present any chemical analysis of the water, which was left for future work.

Mukhopadhyay et al. [104] use a twin-hull catamaran named ASV-Victoria to perform autonomous surveys in regions polluted by crude oil. They focus on developing a controller to enable the robots to follow lines and curves and maintain formation collectively while measuring reminiscent crude oil along their paths. The robustness of control of both USVs was assessed at Grand Isle, Louisiana, where control challenges were reported due to wind and currents. Dalgleish et al. [94] use a Wave Glider equipped with a multiple channel hydrocarbon detection sensor for oil spill monitoring. The developed USV has a Turner Designs C3 optical sensor for measuring crude oil and an AquaTracka deep-ultraviolet fluorometer for measuring Polycyclic Aromatic Hydrocarbons (PAH), to determine the boundaries and origin of the identified surface slicks. Guerrero-González et al. [101] perform oil spill monitoring using a multivehicle system based on a USV combined with a UUV named BUSCAMO-Oil. The two vehicles are connected by a cable, allowing them to share hardware and software. Each vehicle is equipped with a C3 submersible fluorometer with three optical sensors to detect crude oil, refined fuel, and rhodamine. In this system, while the USV creates a map of the extent of the oil spill on the surface, the UUV creates a map of its extent in depth. Thus, the system can draw a precise map of the oil plume, adding information on spill location, volume, extent, direction, and speed. Vasilijevic et al. [93,107] use a heterogeneous robotic system composed of UUVs, USVs, and UAVs to deliver timely information on sub-surface hydrocarbon concentration. In this system, UUVs measure the hydrocarbon concentration while a UAV does an initial survey over a wide area and a USV performs the acoustic localization of underwater agents. Moreover, USVs and UAVs sense the surface and serve as communication links to make the collected data available in real time to a remote ground station. Tests involving control and localization were performed in Biograd na Moru, Croatia and Cartagena, Spain, where the Platform for Dynamic Positioning (PlaDyPos) USV was used to correct underwater positioning of the Light Autonomous Underwater one-man-portable Vehicle, LAUV-LUPIS.

Liu et al. [105] describe the overall scheme of USV-based laser fluorosensor system for oil detection, which consists of a shore-based terminal and a laser fluorosensor mounted on a USV. The laser fluorosensor system sends the collected data to the shore-based terminal by wireless communication. Tests with the laser fluorosensor were performed in a river to measure different targets, such as river water and oil.

A BP/GoMRI-sponsored project was started to investigate the feasibility of using Passive Acoustic Monitoring (PAM) and USVs to help in the estimation of water-life damage after environmental disasters. In that project, Ziegwied et al. [108] test two USVs, C-Enduro and C-Worker—capable of reaching approximately 3 knots and powered by using a combination of solar-, wind-, and diesel-powered engines—during ten days in PAM tasks.

#### 4.1.2. Oil Spill Tracking

Traditionally, oil slick location and trajectory are tracked using remote sensors on satellites and aircraft, such as ultraviolet, infrared, visible band, radar or passive microwave sensors [137]. Monitoring and tracking such pollutants is crucial to creating trajectory models to predict their expansion. Such understanding is essential when creating an action plan to minimize the damage caused by the oil spill over wider areas and over time.

Kato et al. [109,110] describe a project (www.naoe.eng.osaka-u.ac.jp/~kato/project/en/) regarding detection of gas and oil spills in the period of 2011 to 2015 involving a SOTAB-I UUV, an underwater buoy robot, and SOTAB-II USVs, floating buoy robots. In this project, they perform autonomous tracking and monitoring of spilled plumes of oil and gas using the SOTAB-I connected to multiple SOTAB-II USVs. While the SOTAB-I collects rough data on physical and chemical characteristics of plumes, consisting of spilled out oil and gas, SOTAB-II USVs track the spilled oil on the sea surface. Also using SOTAB-I and SOTAB-II, Senga et al. [111,112] perform experiments in Biwa Lake and Osaka Bay, Japan, to verify the effectiveness of sail and sail control for tracking oil plumes. Experiments indicate that once the buoy is dropped into the oil slick drifting on the sea surface, it sets out to drift with the oil slick using the wind force by effectively controlling the sail size and direction. In 2015, Rathour et al. [113,114,138] changed SOTAB-II to a yacht-shaped USV that can track spilled oil on the sea surface, using data supplied by onboard sensors to control rudder angle and sail area for navigation. The new USV takes advantage of the wind to move on the oil spill surface.

Fahad et al. [117] perform plume tracking using fluorometers sensors. The authors performed experiments in Oahu, Hawaii, using an in-house twin-hull catamaran USV, in a site diverse enough to capture most environmental conditions that typically affect the development of a plume. Data concerning the plume dispersion were collected to improve tests in simulators, such as the one previously tested by Fahad et al. [115].

#### 4.1.3. Oil Spill Ensnaring and Removing

When an oil spill occurs, the very first action is to stop pollution at its source to mitigate the adverse environmental effects of an oil spill. Subsequent actions can be containment, recovery, and disposal of the oil. Research efforts are being focused on the development of technologies to remove the oil in situ, minimize operational time, and protect the health and safety of the cleaning crew [123]. A variety of strategies have been developed to cleanup the pollutant and thus, minimize the extent of the environmental impact of oil spills. Ventikos et al. [139] divide conventional cleanup strategies into three groups: natural degradation, where no action is performed apart from monitoring the movement of the spill; mechanical cleanup methods, which include containment and recovery of oil that remains on the sea surface by using barriers/booms or skimmers; and chemical methods, which use dispersants to reduce the interfacial tension between oil and water or other agents such as emulsion breakers, gelling, and sinking agent. We refer the reader to a thorough description of oil spill response methods (e.g., mechanical, chemical, etc.) and corresponding oil response equipment (e.g., booms, skimmers, etc.) in [139].

An application of USVs in *mechanical cleanup methods* includes a team of unmanned ships towing a boom, creating a mechanical barrier capable of controlling the motion and spreading of the floating substance. The idea is that once the operation starts, the USVs tow the boom towards the objective, minimizing the towing effort. Near the objective, the USVs deploy the boom in the water and advance. As they get closer, the fleet of USVs moves in formation to confine the spill, which may then be transported by the formation towards a given destination [125]. Such an approach requires resolving many challenging technical problems such as planning, coordination, communication, cooperation, and navigation of both vehicles.

Arrichiello et al. [118,119] study the cooperative control of two USVs performing a caging and transport mission on the water surface. A flexible floating rope connects the two vehicles with the purpose of capturing the floating oil from a given location and transporting it to a designated position. Experiments to test the coordination control strategy of the USVs to accomplish the caging mission were executed in the Echo Park Lake in Los Angeles.

Pereda et al. [121] also perform oil spill confinement using two USVs towing a boom. Their approach includes a mathematical model of the system and a navigation system based on the Null Space Based behavioral control. The focus of their work is to study the navigation tasks that need to be imposed for a proper towing of an oil spill confinement boom. Later, Giron-Sierra et al. [124,125,140] study the control and coordination needs for the use USVs with autonomous control when automatically towing a boom. The core idea is to provide the algorithm with the area where the oil spill occurred, and the vehicles would automatically try to contain the oil spill. Experiments were performed using two scaled USVs and a ground station, in a lake near Madrid, Spain, to test a parallel formation and the performance of USVs when towing a boom. The results show this method is feasible, provided that some specific criteria were satisfied concerning feedback control and geometry (V-shape) of the boom.

Kim et al. [95] present a robotic system for environmental disaster response that incrementally forms a chain around the floating contaminant. The proposed system uses several USVs to surround and contain the pollutant by docking on each other, in a similar way containment booms would behave. These USVs are connected, floating on the water surface to form a physical barrier to contain the floating contaminant. The system has the potential to work autonomously or being controlled by a human operator. They test the effectiveness of the docking method and initial tests involving the containment of the contaminant.

While booms can be used to control the spreading of oil by confining it to a specific area, skimmers are used in *mechanical cleanup methods* to recover the oil from the water surface. Such robotic system reduces the enormous effort involved in manual skimming operations. Boulougouris et al. [122] and later, Kakalis and Ventikos [123] examine the behavior of a robotic swarm concept for the active confrontation of oil spills. Their system consists of some identical autonomous robotic units that recover oil mechanically employing skimming brushes and can communicate with each other. In a similar approach, Bhattacharya et al. [120] use two USVs towing a floating rope to improve oil skimming by increasing the containment area. Skimmer booms are modeled as a flexible, floating rope of constant length and as a discrete segment model. Equations governing the rope dynamics are derived and tested through simulations trying to maximize skimming efficiency. Experiments to verify the dynamics of a flexible rope being pulled by two USVs are performed using OceanScience QBoat-I hull USVs in Echo Park Lake, Los Angeles. Adapting an approach already in use by manned surface vehicles to USVs, Wang et al. [127] have devised a USV named “HaiTeng 01” that can move at 40 knots with an inclined plane skimmer at the front of the vehicle to collect oil from the water effectively. Unlike most USVs presented in this work, HaiTeng 01 has the capacity to store up to 1000 liters of spilled oil. Experiments to investigate the influences of the oil recovery apparatus located on the front deck in the performance of USV at high speed were performed in Shenzhen Bay, China.

Wang et al. [126] develop a Porous Unmanned Ship (PUS), a USV with aligned ZnO nanorod arrays on the surface of the porous stainless steel wire mesh with properties of superhydrophobicity and superoleophilicity. Hence, when the PUS contacts with the oil, it is quickly pulled toward and penetrates the PUS automatically. Experiments are performed showing that the superhydrophobicity and low water adhesion force of the mesh surface endow the PUS with high oil/water separation capacity (above 94%), illustrating the importance of the vessel design for the given task.

#### 4.1.4. Oil Spill Simulation

As in situ experiments are costly and time-consuming tasks, environments which simulate the real world can be an asset for oil spill DM. On the other hand, accurate and computationally efficient simulations of ocean pollution are critical to the continued development of new techniques for autonomous environmental monitoring [117]. Therefore, research involving the simulation of environment and methods to deal with oil spills are attracting a significant number of researchers.

Fahad et al. [115] present a simulation of a single robot that performs oil plume tracking. This simulation aims to perform the validation of the controller as well as test the robustness of the control using complex probabilistic environmental models. Later field experiments are performed using a dying marker to collect further data to improve future simulations [117]. Li et al. [141] expand this idea and simulate plume tracking algorithms using multi-robot. In contrast to existing work purely relying on gradient measurement, the transport model of pollution source is explicitly considered in tracking control design.

Saldaña et al. [116] propose a decentralized coordination method that allows multiple robots to efficiently sample and predict the behavior of environmental boundaries, such as the ones generated by oil spills. In their method, the robots first identify the boundary of the substance. When the robots reach the boundary, their primary task is to accurately follow a static or time-varying boundary by maintaining the robots equidistantly distributed along the curve. The method can estimate the shape of the boundary using the collected point-wise measurements. They test their method in a small-scale experiment using multiple USVs in a pool.

### 4.2. Biological Hazards

Although the microscopic planktonic algae are critical food for filter-feeding bivalve shellfish (e.g., oysters, mussels, scallops, clams), in some cases, the proliferation of plankton algae (so-called “algal blooms”) can have an adverse effect to aquaculture, fisheries and tourism operations [142]. There are over 5000 algal species of documented phytoplankton species. However, about 50 of them can be harmful to the environment, due to the toxins they produce. Such toxins can harm and contaminate the water life and even kill a human [143]. Marine algal toxins are responsible for more than 60,000 intoxication incidents per year, with an overall mortality rate of 1.5% worldwide, and for die-offs of fish and shellfish and have been implicated in the mortality of marine mammals, birds, and other animals dependent on the marine food web [144]. The problem of Harmful Algal Bloom (HAB) is so severe that when it occurs in the United States or European Union, it results in sampled saxitoxin concentrations more than 80 μg per 100 mg of molluscan tissue. Commercial and recreational fishers and growers are then precluded by law from harvesting and selling shellfish [143].

Due to the threat to freshwater ecosystems, the occurrence of massive development of noxious cyanobacteria (blue-green algae) is increasing. Such algae cause a variety of harmful impacts on the aquatic environment, since they produce toxic substances [145] and reduce the dissolved oxygen in water due to the decomposition of extensive amounts of organic material. To identify such toxins in HABs and avoid human exposure to hazardous conditions, researchers are considering USVs for performing HAB monitoring tasks. Table 4 presents the work that use USVs to conduct environmental monitoring for HAB identification, where *NR* means “Not Reported” to data not reported by the authors.

Higinbotham et al. [146,147] introduce the development of a new long-duration solar-powered USV known as the Ocean-Atmosphere Sensor Integration System (OASIS). The platform intends to function as a low-cost, long-duration, reusable, navigable, open maritime platform focusing on the collection of measurements at the ocean-atmosphere boundary layer. It is equipped with sensors for water measurements (e.g., temperature, salinity, depth, colored dissolved organic matter, chlorophyll, phycoerythrin, and rhodamine) and meteorological measurements (e.g., wind, barometric pressure, relative humidity, pressure, and temperature). One of the objectives of the OASIS is to contribute with the development of new systems and techniques for understanding and monitoring of HAB.

Dolan et al. [148] suggested HAB characterization with a fleet of USVs. In that work, a Sensor Web-relevant system called the Telesupervised Adaptive Ocean Sensor Fleet (TAOSF) enables the in situ study of surface and sub-surface characteristics of HABs. The system is composed of OASIS USVs as well as the land-based control and communications infrastructure. The platform also provides a mast-mounted meteorological station enabling acquisition of atmospheric measurements including barometric pressure, air temperature, relative humidity, wind speed, and wind direction. Later [149], experiments were performed in the Chesapeake Bay area using OASIS platforms and autonomous kayak to test and validate sensors as well as assemble and to analyze HAB-related data.

Elfes et al. [151] describe a Multilevel Autonomy Robot Telesupervision Architecture (MARTA) for multi-robot science exploration. The telesupervised architecture with a combination of unmanned systems involving the OASIS platform is used for the detection of HAB. Researchers designed a technique which uses an USV for monitoring and tracking harmful Dinoflagellate Cochlodinium polykrikoides, responsible for red tides that can kill fishes, damage coral reefs, and interfere with desalinization plants [159]. Besides the use of a USV, experiments are carried out using aerostat: an unmanned, lighter-than-air unpowered blimp on a tether. It carries an avionics package with a recording GPS, barometric altimeter, magnetic compass, serial data link, wide-angle color camera, and transmitter to view how an algal bloom is moving and dispersing in the water, and how the OASIS USVs are responding to this process. Extending the idea of Elfes et al. [151], Low et al. [152] develop a new Robotic Sensor Boats (RSB) to integrate to MARTA. The new boats are developed with the primary focus on the sensing and navigation requirements, with the hull as a roto-molded recreational kayak that is fitted with a ducted thruster.

Pereira [150] describes the conversion of a remote-controlled boat (Roboduck-II) into a USV capable of navigating relatively complex water bodies including lakes and marinas. The USV has GPS, a navigation system encompassing a simple stereo vision-aided framework for obstacle avoidance. Roboduck-II is used to conduct biological sampling to monitor HABs. Tests were performed at Redondo Beach, California, USA, where the sensor package was lowered up to 5 m to collect samples.

Seo et al. [159] develop a tracking path planning algorithm for the detection of HAB. The algorithm is tested in simulation, where they conclude that when a robot meets the first occurrence of Cochlodinium red tide, it is possible to track them and provide useful real-time environmental information. Also in simulation, Arzamendia et al. [160] aim to use USVs for detecting and monitoring the cyanobacteria in the Ypacarai Lake, in Paraguay, and visiting a ring of beacons at the shore of the lake for data delivery. This problem is modeled as a special case of the Traveling Salesman problem, where the distance should be maximized and not minimized. However, instead of cities, the problem is seen as visiting the equally spaced beacons along the border of the lake. The path planning approach uses a genetic algorithm to try to find an optimal solution. The approach is tested in simulated environments.

Hitz et al. [158] describe the design of a USV equipped with limnological sensors to collect physicochemical data and monitoring HAB (e.g., Planktothrix, a significant producer of hepatotoxic microcystins, which can harm the liver). Results from one year of missions over Lake Zürich containing both HAB and temperature measurements, where authors argue that spatial resolution should consider different sampling depths since HAB can be found even at 20 m of depth. The authors also point out that the amount of HAB varies over time and space requiring several measurements over the year.

Zhang et al. [161] proposed an approach using a gliding robotic fish, which is a hybrid of underwater gliders and robotic fish. The gliding robotic fish named Grace is used to detect HAB not only at the surface, but also sampling multiple water columns, providing a better assessment during field experiments involving the sampling of harmful algae concentration in the Wintergreen Lake, Michigan. As Zhang et al. affirm, sampling from multiple water columns is important not only in the monitoring process but also in facilitating mechanistic modeling and understanding of the development of HABs.

With a marine microscope imaging system with automated cell characterization capability attached to a solar-powered Wave Glider, Ziccarelli et al. [162] acquire crop and geo-tag phytoplankton images. Such images allow a near real-time detection of harmful algal species as well as the calculation of their population density. Data is then sent to land for analysis via mobile phone or satellite communications. The collected data provides input for computational models to advance the understanding of phytoplankton and allow better forecasting of HAB events.

Other USVs containing HAB detection capabilities include the solar-powered Lake Wivenhoe USV [153,154,155,156], which is a twin-hull catamaran capable of navigating in complex inland water reservoirs and measuring a range of water quality properties. The 16ft long solar-powered catamaran is also capable of collecting water column profiles while in motion.

### 4.3. Radioactive Hazards

As mentioned earlier, disasters usually are caused by natural or man-made events or a combination of them. They can spread rapidly, causing severe environmental damage with unknown contamination causes (e.g., nuclear pollution accidents, such as the Japan nuclear disaster in 2011). The 2011 incident at Japan’s Fukushima nuclear power plant resulted in one third of involved rescue workers being subjected to high levels of nuclear radiation, facing a higher lifetime risk of cancer, while hundreds of thousands had to be evacuated from the Fukushima area [163]. In such emergencies, various professional and volunteer rescue workers collaborate, where they are subject to an extremely harsh and dangerous environment with high personal risk. Hence, recent research has explored the feasibility of using robots for conducting activities in harsh radioactive environments.

Given the proximity of many nuclear reactor sites to large bodies of water, the deployment of USVs for monitoring or even support can be a natural fit [164,165]. In particular, after the accident in Fukushima earthquake/tsunami, it became clear that radiation from reactors can indeed leak to the body of water in case of extreme events, endangering life and compromising rescue operations.

Nuclear Biological Chemical sensors and robots, including an AEOS Marcy USV [166] have been integrated and tested in a radiological forensics field exercise in 2013. An international maritime interdiction operation experiment was conducted in the San Francisco Bay involving the connection of two radiation detection sensors to a mesh network consisting of multiple sources, including a Seafox USV [167] which was used for a drive-by search. The collaborative environment where experts can assess information from numerous locations/events simultaneously has shown to be favorable to detect radiation sources in military and homeland security operations. The authors mention management and networking issues, which must be addressed to pass control to the Coast Guard. We highlight that such a strategy could be extended to disaster sites such as Fukushima to detect radiation leaks in the ocean.

Wilde et al. [168] designed a USV for radiation detection, heat map creation, and source localization in a controlled test environment in a “disaster city” where they deployed a Cesium-137 source. The study also involved the discovery of a proper path in detecting such source [169], considering that USVs cannot perform hard turns during raster monitoring of certain areas. Matos et al. [170] detail its exercise from euRathlon’s 2015 maritime SAR competition, employing multiple robots, including a USV (ROAZ), which can deploy other unmanned systems. They argue that the use of USVs for a rapid initial search and subsequent use of UUVs is powerful in scenarios such as the Fukushima disaster, to narrow down the search. The combination of USVs and UUVs is also explored in [171].

Recently, researchers went back to the Bikini Atoll, where tests for the development of the atomic bomb were performed during WWII, to understand how nukes and accidents such as the Fukushima Daiichi have and will impact the environment [172]. They used a Jetyak USV, equipped with a sensor to sample water and detect radioactive compounds, to collect water samples from the area. The idea was that as the Jetyak floated across the lagoon, the sampling device would pump water through cesium extraction “sponges”, to measure levels present in the lagoon.

## 5. USV Applications for Preventive Maintenance and Disaster Response

This section presents USV applications for the phases before a disaster (preparedness and mitigation phases), specifically structure inspection for preventive maintenance (Section 5.1), as well as applications for disaster after-effects (response phase), specifically structure inspection for damage assessment (Section 5.2) and search & rescue of people in water (Section 5.3).

### 5.1. Structure Inspection for Preventive Maintenance

Robotic inspection has gained visibility in civil engineering [45], an area not traditionally connected to robotics. Its applications with regards to disasters can be classified by the management phase in which they occur: in the preparedness and mitigation phases, before a disaster, structure inspection is performed to aid preventive maintenance of buildings and infrastructure, potentially minimizing damages and losses in the event of a disaster.

Historically, USVs for preventive structure inspection was first proposed to the application of bridge scour assessment, a task typically performed by a team of divers and engineers, using manual cameras, human vision and touch [173]. According to the United States Department of Transportation [174], scour is the erosion of stream-beds or bank material due to water flow from natural currents, or debris brought by disaster events (e.g., storms, tsunamis or floods). When it happens around a pier, bridge, or other manned structure, it can open gaps in the bed supporting the structure, risking its collapse and turning it into hazardous environment for the inspection crew. Mueller and Landers [175] proposed a USV which successfully assisted teams performing bridge scour assessment in six flood events.

Apart from bridge scour assessment, USVs are also used to verify structural health in above-water or partially submerged constructions, with 3D reconstruction [176,177,178,179,180,181,182], for example, where USVs collect sensor data from a structure (e.g., camera images, sonar distances), reconstructing the structure for offline inspection. In this application, Kurniawati et al. [176] use a USV to capture 3D LiDAR sensor data of oil rigs and dams to perform the subsequent reconstruction for preventive inspection. The authors tested the USV in the Singapore Strait, where several structures were reconstructed from the captured data.

One of the challenges for accurate 3D reconstruction is that precise localization is necessary when collecting data, typically not available using only GPS sensors, as shadowing effects and line-of-sight occlusion significantly affect the localization accuracy. Leedekerken [177] solves this problem with a new framework for 3D SLAM, which combines data from above and below the water surface (i.e., a heterogeneous environment). The framework is integrated and tested in a USV capable of robust mapping and reconstruction of 3D marine structures.

Likewise, Papadopoulos et al. [178,179] use USVs to scan both above and below the water surface, to perform 3D reconstruction of partially submerged marine constructions. The solution uses the Iterative Closest Point (ICP) algorithm, with data from a Velodyne LiDAR (model HDL-64E S2) and a BlueView side-scanner sonar (model MB2250), to reconstruct the above and below-water parts of a jetty in Selat Pauh, a small island on the Singapore Sea. The authors note that powerful water currents change the unmanned vehicle’s roll and pitch motions, sometimes causing outlier data in the LiDAR scans, which must be previously filtered.

Han et al. [180,181,182] perform 3D reconstruction of bridges and semi-submerged offshore platforms. In [180,181], the authors use the ICP algorithm along with data from cameras, Inertial Measurement Units (IMU), GPS, and LiDARs installed on a Kayak-based USV, to reconstruct above-water bridges in the Bang-Dong reservoir, in Korea. As a limitation, the 3D reconstruction is sensitive to navigation and positioning errors, as GPS accuracy is affected below bridges. In [180], the authors fuse data from a USV’s sensor array, including 2D and 3D LiDAR, IMU, GPS, and underwater sonar sensors, to reconstruct the hull of a semi-submersible offshore platform in Okpo, Korea. To avoid the reconstruction sensitivity to localization errors, the USV performs local navigation, relative to the planar hull structure, dismissing the need for high GPS accuracy.

More recently, Shojaei et al. [183,184] used USVs to identify cracks and other structural problems in concrete seawalls and retention ponds, with computer vision methods. The authors prototyped a small and low-cost USV, mounted with a camera which captures images from the concrete structures, performs segmentation (i.e., separates water from concrete), and analyzes the concrete regions for the presence of cracks and water deterioration.

Lindemuth et al. [79] proposes a novel solution to structure inspection, using a “marsupial robot team”: A Sea-RAI USV hosts an UAV, used when necessary. The authors verify the robot team usefulness in several applications, such as littoral inspection, environmental monitoring, port security, and preventive infrastructure maintenance. The tests were performed in incremental complexity, at Bayboro Harbor and Pensacola Naval Station, both in Florida, United States. The authors identify that for marsupial platforms to be effective, better control interfaces and robot autonomy are needed.

### 5.2. Structure Inspection for Damage Assessment

In the response phase, after a disaster has occurred, USVs can be used to perform damage assessment in buildings and infrastructure. The goals of this application are to perform structural inspection and evaluate the constructions’ post-disaster structural integrity, identifying dangerous areas for response crew, as well as mapping debris and locating safe access routes to affected zones. However, few works have real-world deployment for this application, as it must take place after a disaster.

The first known deployment of USVs for damage assessment was in 2005, three days after Hurricane Wilma’s landfall [19,61,62,83]. The Center for Robotic-Assisted SAR (CRASAR), along with the Institute for Safety Security Rescue Technology (iSSRT) used a USV and UAV team to assist response crews in verifying the structural integrity of Marco Island’s seawall and piers, as well as to locate submerged debris and define safe lanes for sea navigation. In this deployment, the authors validated the suitability for USVs in DM, identified cooperative USV-UAV strategies (i.e., USVs may provide external view for situation awareness, spot areas to be inspected and serve as communication relays), and identified a UAV deployment pattern, where short and localized flights, made to take advantage of line-of-sight, are preferable over a single continuous fly-over.

In 2008, CRASAR also assisted in the response phase of hurricane Ike [62,63,64], successfully deploying a Sea-RAI USV to inspect the structural integrity of the Rollover Pass Bridge in Texas, USA. The deployment missions were: (1) To evaluate the utility and performance of the USV in inspecting the bridge’s submerged structure; (2) To map the hurricane’s debris field. In the second mission, the Sea-RAI was deployed three times, using an acoustic camera to obtain images of the bridge substructure and debris surrounding its pliers—which were analyzed by the response crew to verify the bridge’s structural integrity.

The deployment team identified challenges in three areas: USV control and navigation, human-robot interaction, and data uncertainty. First, swift water currents limited the times and duration of the USV missions and required tethering of the USV. Operation near and under bridge also produced GPS loss and errors (1% away from the bridge and 22% near or under it), which required teleoperation of the Sea-RAI. Second, the deployment confirmed the need for multiple displays for different information and reinforced USV payload. As different specialists were involved in the disaster response team, not all information from the robot is useful for everyone, which calls for multiple customizable displays. In the broader context of human-robot interaction, the USV’s payload must be robust to withstand the water force, as it knocked the acoustic camera out of alignment during operation, confusing the operators and leading to coordination challenges between team members. Finally, uncertainty in the data from the acoustic camera due to shadows and differing viewpoints presented challenges for the accurate understanding of submerged structures.

### 5.3. Search and Rescue

After natural disasters, there may be survivors adrift in the water or groups in life-rafts. Failure to rapidly respond to disasters and rescuing survivors in the water can often lead to prolonged suffering and death. When performing SAR in this context, USVs’ capabilities can be a powerful asset. A USV can cover a wide area fast over the water surface, carrying survival kits, or providing emergency communication infrastructure, while also performing coordinated operations along with other unmanned systems [185]. General-purpose USV research often mentions SAR as a motivation for autonomous USVs. However, few works truly address the problem of SAR directly.

#### 5.3.1. USVs for SAR

Preliminary work on SAR remounts to the second world war [24,28]. However, documented research often mentions USVs for SAR starting in the late 1990s. In 1995, the University of Rostock developed in 1995 Rescue Dolphin, a USV for SAR of people in distress [24,186]. The system automatically triggers an alarm in case of people overboard, activating the USV which rapidly moves toward the person adrift, securing the victim until the rescue by ship. According to Bertram [24], in the last decade, a Canadian company called (ISE) developed two vessels for SAR: the Seal USV, for demonstration purposes to Canadian Department of National Defense (DND); and the SARPAL, which can be deployed from the air. Motwani [28] mentions the Sterling USV, which can be used for SAR as well, along with several CRASAR initiatives involving USVs for DM.

SAR operations using USVs often involve the use of different vision sensors, some background subtraction strategy, along with some object detection & tracking technique [187]. However, the scope of this section is not to address video and image processing techniques—interested users are referred to [187] as a starting point. In the same way, the scope of this work is also not coverage path planning or GNC, where relevant work and surveys are abundant and presented as background work— [188] is a recent and specific survey on coverage path planning. Below we address such works involving SAR and USVs directly.

Wang et al. [189] devised a multi-purpose USV, under the support of Innovation Program of Shanghai Municipal Education Commission, capable of performing water sampling, hydrographic surveys, and SAR missions. The USV is equipped with GPS and an infrared camera with a range of 100 m at night. The USV can carry more than 100 Kg of payload, to carry a person or lifesaving appliances to a castaway.

Regarding detection of survivors in the water, Govindhan et al. [190] describe a USV equipped with an Arduino-based system for human detection. There are also preliminary experiments emulating the search of human bodies after disaster events, conducted by [191,192,193]. Lee et al. [193] devised a robust method for the detection of submerged bodies using ultrasound, which is difficult in turbid scenarios. The task is challenging, since underwater ultrasound image may not straightforwardly be converted into geometric shapes, mainly due to heavy noise on its characteristics. The authors make use of a Convolutional Neural Network and the Caffe framework to identify submerged bodies with good results in tests performed in a Lab pool.

Kurowski et al. [194] developed a satellite-guided SAR system, to be used in special ships and offshore platforms in case a person falls overboard. It consists of three main components: (1) A vest with an Automatic Identification System (AIS), worn by every crew member of a ship or offshore platform; (2) An autonomous twin-hull catamaran USV, referred to as the “rescue vessel” (described in detail in [195,196]); A satellite-aided control station. When a person falls overboard, the AIS vest activates upon contact with the water, and the SAR process starts. The vest uses a differential GPS to determine the person’s position and broadcasts it to the control station as an AIS message. Upon receiving this message, the control station deploys the rescue vessel, which autonomously navigates to a minimum safe distance from the person. A human operator in the control station then assumes manual control, approaching a salvage position based on video information from the USV. After the person is rescued, the rescue vessel and control station ship navigate toward each other, at which point the USV is picked up by the control station ship, completing the rescue scenario. The authors tested USV for maneuvering, swell and free fall tests in the swell basin of the Technical University of Berlin, proving its feasibility at sea, with rough waves. A successful test of the full SAR system was performed in the port of Rostock, Germany.

The EMergency Integrated Lifesaving lanYards (EMILY) (http://emilyrobot.com/) [66] was the first robust teleoperated USV designed for water rescue applications. EMILY was used for rescuing migrants in the Syrian crisis and is currently in use by the Los Angeles County Fire Department (LACoFD) Baywatch. The USV was modified to include a Pixhawk controller which implements waypoint navigation, return to launch, as well as an improved user interface design. After feedback from different trials, the SmartEmily autonomous USV [197] was conceived. Its current interface incorporates the input information provided by the Castrium Rescue Brigade and LACoFD Baywatch and was tested at the DHS CAUSE V Exercise in Bellingham, Washington. Schofield et al. [198] propose a potential fields-based algorithm to gradually slow down EMILY, as it approaches the location of drowning victims, to keep them safe and facilitate first-responder tasks.

A major endeavor involving institutions from ten European countries and funded by the European Community’s Seventh Framework Program (FP7/2007-2013), called Integrated Components for Assisted Rescue and Unmanned Search (ICARUS) (The ICARUS project budget was in the order of 17.5 million Euros [19].), is focused on large-scale maritime assistive robotic tools for SAR operations [199,200,201]. The ICARUS initiative designed and tested several USVs for SAR: among them, two small Unmanned life-raft robotized CAPsules (UCAPs) [202,203,204]. The first UCAP [202] uses conventional propellers and can be deployed from larger vessels, including an automatic deployment and inflation system designed for a life-raft for four people, in compliance with the International Convention for Safety of Life At Sea (SOLAS Convention—http://www.imo.org/en/About/Conventions/ListOfConventions/Pages/International-Convention-for-the-Safety-of-Life-at-Sea-(SOLAS),-1974.aspx) (SOLAS). The second is the SWIFT USV [203], designed to operate in shallow water and surf zones, also carrying a life-raft for four people, but incorporating lessons learned from the first UCAP. It is smaller and lighter than both EMILY and the first UCAP, but faster than the latter since it uses a water-jet propeller (like EMILY, trading efficiency for speed). Furthermore, it includes a robust pose estimation framework, with better estimators and hardware to reduce magnetic interference. Still, its life-raft deployment system was not tested.

Later in 2016, a third UCAP was designed [204,205] with slightly larger dimensions, incorporating features from the previous two designs. However, a comparison with previous versions of the UCAP is not provided. During the Robotic Exercises 2014 (REX’14) [206] the first two were deployed from the ROAZ USV [76], a larger USV. The ROAZ USV can map an area before the intervention of an UUV, or rapidly get to a SAR site carrying and deploying UCAPs. Field experiments in different locations were performed using the ROAZ USV combined with color and infrared (IR) cameras [207], for finding a person at sea and detecting obstacles. Their strategy encompasses the detection of the horizon line using an edge detector combined with a Hough transform–the search for casualties is performed below that line using the USV IR camera. The infrared camera contrast is improved, and a stereo pair of IR and color cameras is performed to determine de 3D position of the person at sea. The team presents evidence that the IR pattern of a person at sea is considerably salient—even at a distance and when the subject is wearing a swimsuit covering most of their body. Among the relevant information collected, the ICARUS initiative has raised the importance of a 360${}^{\circ }$ cameras and 3D rangefinders for SAR. Beyond ROAZ and the UCAPs, there were other USVs tested in REX 2014, such as the previously mentioned SWIFT, and ZARCO [208], a USV designed for assisting UUVs in rivers and estuaries.

#### 5.3.2. Heterogeneous Teams of Unmanned Systems for SAR

Detection and tracking of people and life-rafts adrift at sea is often performed by airplanes and cameras, which can offer a wider field of view of the sea surface, in comparison to those of ships and USVs at the sea surface. However, this process is often tedious and challenging due light reflections at sea, intermittent target occlusion, the distance from the plane to a target and its size relative to this distance. Therefore, the automation of such process is currently a research topic, where vision systems are used to detect and track survivors [209,210]. More recently, researchers are investing in effective combinations of heterogeneous unmanned systems, such as UAVs and USVs [211] to further automate SAR operations at sea.

The ICARUS project stands out as the leading initiative focusing on SAR in marine environments [205]. Notwithstanding, ICARUS’ resources encompass robust unmanned systems designed for air, land, and water SAR operations, and training exercises tested its operational validation, involving different scenarios such as shipwrecks, where a team of robots presented effective results while detecting and helping people on the water [201]. The primary goal of the project is to enable first-responders with a team of unmanned systems, along with management tools to enable faster and more effective SAR operations [21].

In [212], a USV-UAV team is used to detect castaways using a particle filter computed in the USV, as it has higher computational power than the UAV, which in turn only provides aerial images as the filter’s input. The system uses Artificial Neural Networks to compensate water and wind disturbances, predict the castaway’s position, and find and track them. Unfortunately, the system is only tested in simulated scenarios. A combined UAV-USV decision-making strategy for maritime rescue, based on Bayesian Network, is proposed in [213], which also features only simulated experiments. Rafferty and McGookin [214], designed an AUV-USV rescue system to search for survivors in the water efficiently. The system is comprised of one USV which can launch four UAVs helicopters which search for survivors by measuring the temperature of each point visited in the search space using an IR camera. The authors conclude that a particle swarm optimization algorithm is superior to a random search strategy in 20 simulated experiments.

Cooperation between EMILY and a Fotokite UAV [215] was proposed in [216], to assist drowning victims by determining EMILY pose from pre-processed video feeds from the UAV, using different video strategies. Two methods were used for position estimation: (1) Color thresholding, erosion, dilatation, and blob detection; (2) Histogramming, back-projection, and CamShift. The orientation was estimated by fitting a minimum area ellipse over the blob and finding its greater axis. Later, a different camera stabilization method was developed [217], to correct camera pose errors due to wind and motion of the Fotokite, which is validated in a set of trials. Xiao et al. [218], presented several tests in four outdoor scenarios using EMILY and the Fotokite, where the UAV provides first-responders with a top view of the scenario, covering a large area to guide the USV autonomously to victims without the requirement of manual operation, freeing the rescue team to other tasks.

In [219] the authors argue that the use of rotary wings UAVs, which prioritize camera motion, can provide better situation awareness to operators in disaster scenarios by increasing the amount of time USVs are visible to the UAV when compared to traditional motion using fixed-wing UAVs. Recently, Dufek and Murphy [220] aimed to rigorously define the sub-problems and assumptions about the control of USVs using UAV top-view information. They provide an in-depth theoretical background for each sub-problem and define the theoretical lower-bound limits for their solutions. They conclude that even though localization precision cannot be improved, motion planning can be the focus of future research involving the cooperation of UAV-USV for SAR.

An approach combining the Pelagi USV, a 4.5 m Nacra catamaran, and the Vigil R6-WT UAV (a six-rotor vehicle capable of take-off and landing in water) was devised for SAR, to provide basic life-support kits and to provide shipwreck survivor position information to the rescue team. The USV is equipped with a helipad, where the UAV can land and recharge its batteries. Experimental tests demonstrate the use of a 360${}^{\circ }$ field of view and IR cameras to detect survivors using the USV [221], as well as the UAV landing in water and detecting survivors successfully.

The Cognitive Autonomous Diving Buddy (CADDY) is an FP7 project (http://www.caddy-fp7.eu.) whose objective is to assist human divers using unmanned systems, namely USVs and UUVs, and other innovative technologies. The key idea behind the CADDY project is to use a UUV as a diver companion, taking photos and other tasks such as guiding the diver and bringing objects to the surface. One version of the system encompasses a MEDUSA USV [222] to determine its localization, the localization of a MEDUSA UUV (the diver buddy) and feedback it to the UUV and the diver [223]. The CADDY project also devised a way to reconstruct and track diver poses using 17 inertial sensors over the diver’s body [224] fused with an analysis of stereo cameras from the UUV using a Long Short-Term Memory Recurrent Neural Network (LSTM-RNN) algorithm [225], to increase the understanding of diver behavior and to detect possible risks to the underwater diver. Mišković et al. [226] propose a way to track divers using a PlaDyPos USV, equipped with an acoustic positioning device, directly over the diver. Carefully planned experiments led to a mean error of 1.8 m due to factors such as air bubbles and diver motion uncertainties. The project trials were designed to detect information about a diver potentially in distress. Initial tests [227] included localization, tracking, and diver activity detection, while the final validation [228] presented the warning system for the diver in distress and showed that divers felt safe and comfortable using the system. Divers often are part of disaster scenarios as part of first-responders acting in extremely harsh environments such as the cave where the Thai boys were trapped in 2018, and one diver lost his life trying to save them. For this reason, we deem the CADDY project a fundamental stepping stone to assist SAR divers in the future.

## 6. Discussion

**Figure 3:**
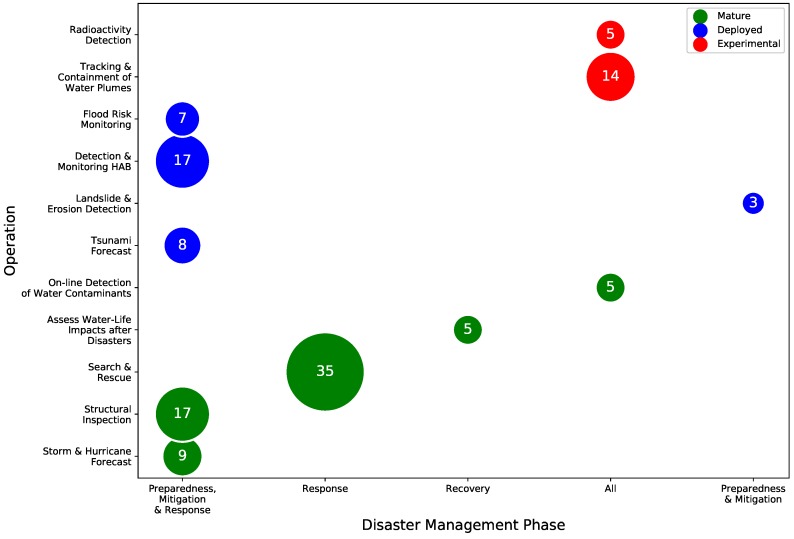
Visual representation for the number of works in Table 5.

This paper surveyed the use of USVs and their role in the DM process. This section presents recommendations for USVs in the DM process. Table 5 presents the current work on disaster robotics involving USVs. We classify each operation according to the DM phase and maturity level, where “mature” means the technology is ready to be used, “deployed” means it was at least tested in the field, and “experimental” means preliminary experiments were performed. Figure 3 presents a bubble chart for the visual representation of the number of works in Table 5, classified by the operation and disaster management phases. The number of works is directly proportional to the size of a bubble, while the colors represent the maturity level for each operation. Please note that the use of USVs for DM is maturing across different applications. Finally, this section presents a series of technological and non-technological guidelines which have an important role in the DM with USVs.

### 6.1. Technological Guidelines

This section presents and discusses other technological (hardware and software) recommendations and guidelines for USV research focusing on Disaster Robotics.

*Connectivity*: Communication is fundamental for the operation and to guarantee that relevant sensor data reaches the operation center on land [229]. Still, communication problems with USVs often occur, since connectivity depends on the environment, weather, and wave conditions. Furthermore, the availability of broadband connectivity is limited in remote areas of the ocean [230] where only satellite communications with limited bandwidth are available. Even in shallow water and obstructed regions, in situations which cannot be handled by the USV alone (e.g., undetected imminent collisions), communication issues can be the difference between saving or destroying the USV, since the operator will not be able to intervene, jeopardizing the integrity of the USV and others. Therefore, communication problems must not be underestimated. Robust delay-tolerant protocols and equipment should always be considered for DM missions. However, as the human interaction with the USV increases, the bandwidth requirements also must increase to provide real-time video and responsive control, among other bandwidth demanding requirements involving USV sensors data [231]. Finally, in disaster scenarios where the fixed infrastructure may be compromised, IEEE 802.11 and satellites may be the best options to implement emergency communications for first-responders and victims.

*Localization*: Bad weather, being near the coast or man-made buildings (e.g., below bridges or next to dams) are all situations where localization problems related to poor GPS signal may occur. Therefore, localization strategies should be versatile in case of GPS problems.

*Situational Awareness*: In disaster sites, one of the primary functions of USVs’ sensors is to provide information about its surroundings to operators [232,233]. For USVs, it may involve the use of 360${}^{\circ }$ cameras, effective underwater and out of water mapping sensors, microphones, and speakers. Such features will be helpful for several operations, including SAR, and detection of hazards in the surroundings of USVs.

*Information Sharing*: The operator must not be the only person with access to real-time information from the USV, including cameras. Information access to responders and experts must be straightforward. Enough bandwidth and an adequate user interface are required to allow multiple simultaneous users.

*DM Fleet*: A fleet of USVs offers advantages over a single USV including configuration flexibility, redundancy, coverage and throughput [234,235,236]. Furthermore, USVs and UUVs are complementary, and their combination can be beneficial for DM. For instance, disaster damage to man-made structures may be above and below the water level and sometimes only accessible with UUVs—e.g., oil spills and bridge inspection. UUVs can take advantage of USV localization and underwater mapping information [237], communications as well as communication capacity. Finally, UAVs can offer a view from the disaster site which is not possible for USVs [61,79,211], paramount for detecting victims [216] or hazards through an upper field of view.

*Docking and Towing Capabilities*: An asset for missions involving heterogeneous marine vehicles is enabling a USV to launch, recovery & docking for UUVs, UAV, or even other USVs. For example, Zhang et al. [73] uses a USV to carry a UAV into the complex stricken area. Then, while the USV approaches the affected area, the UAV takes off from the USV and sends global information about the environment. This way, it is also possible to rapidly carry UUVs to disaster sites and perform underwater tasks as needed. Similarly, USV towing capabilities could be used to tow containment booms [124,125] or vessels in distress [238,239] to help contain further environmental disasters (e.g., oil spills or ship sink) in case of maritime accidents [240]. It may also be possible to connect multiple USVs to form an autonomous containment line to pollutants or a blockade to alien vessels [95].

*Thrusters*: There are objects such as plastic objects, and plants, such as eelgrass, which may damage underwater propellers [102,241]. Furthermore, propellers should not pose a hazard to humans in disaster sites (e.g., drowning victims). Therefore, USV design should consider proper propeller casing to preserve its integrity and prevent injuries to others in need e.g., the UCAP moved from conventional propellers to water-jet ones [202,203,204]. Cruz and Alves [242] argue that sailboats can be effective for both monitoring and disaster response due to the lack of propellers and the potential for power savings. However, the absence and excess of winds may limit the use of sailboats in real disaster response scenarios. On the other hand, Scerri et al. [70] indicate the use of airboats since usually possess a flat-bottomed hull, using an above-water fan to propel themselves forward safely and effectively through shallow or debris-filled water. Finally, thrusters should be strong enough to compensate or minimize river/sea current effects which, in case of flooding, can be stronger than in normal scenarios [90]—which may be problematic for airboats.

*Bathymetry*: Even though bathymetry instruments are essential tools to address many problems, they are prone to errors which depend on sensor limitations and the environment (e.g., up to 30 cm average error were reported in some studies [77]). Performing bathymetry surveys during high tides is a good strategy as it is possible to place the USV as close to the shore as possible to assess regions near the water with risk of collapse to improve the bathymetry results. Furthermore, calm waters are always the best scenario of choice for a survey, since environmental disturbances such as waves and wind may affect instruments.

*Sensor Payload and Threat Detection*: Appropriate sensor positioning must be considered during the design and testing of the USV, to avoid problems while in operation: as seen in [62], the sensor payload must be robust to withstand the water force, especially in the case of underwater sensors since it may damage or knock them out of alignment. Furthermore, a USV designed for DM should ideally be equipped to detect different types of threats, such as nuclear, biological, chemical and even explosive detectors: for example, the CBRNE sensor system, which integrates Chemical, Biological, Radiation, Nuclear, and Explosive sensors [243].

*Load Capacity*: The USV must be able to carry all sensors, batteries, and extra weight, but if the vehicle is expected to work in shallow waters or ebbs, the weight must not be excessive to allow for mobility [90].

*Real Scenario Testing*: USVs must be thoroughly stressed and tested in real-world situations before their actual deployment, risking complete mission failure. Disaster sites can be dangerous both to humans and USVs. Currently, exercises with the navy and disaster missions [167,201] simulated in competitions [168,169,170] are ways to perform such evaluations and operational validation. Schneider et al. [244] argue that SAR scenarios can be used to validate robotic systems. However, in some cases such as those involving extreme hazards, e.g., radiation, it may be more suitable to make use of computer simulations [165] instead.

### 6.2. General Guidelines

This section presents research and management issues that impact the performance of USV deployment in real disaster scenarios.

*Training*: There are many complex aspects associated with USVs, including data fusion & interpretation, vehicle control, and maintenance, which should be considered while using USVs. Ideally, they should be easy to use and deploy. However, a major problem for response teams is that disaster events are sporadic. Thus, there may be a long period between training disaster response teams to the USV use. Therefore, training exercises and competitions are fundamental [170,245,246] to test, prepare, and maintain emergency response personnel readiness, mitigating the long periods where response teams and technology are idle.

*Transparency*: Many disaster-related problems can raise concerns from the population regarding effectiveness to respond to disasters. USVs (and unmanned systems in general), combined with social media, can be valuable assets for real-time disclosure of risk management information. For instance, unmanned systems strategically positioned along the coast can be used to forecast and automatically warn affected populations about extreme weather or HAB beforehand, using social networks.

## 7. Contributions to the Use of USVs for Disaster Applications

This paper is inserted in the context of a research call organized by two Brazilian institutions, named CAPES (Coordination for the Improvement of Higher Education Personnel) and CEMADEN (National Center for Natural Disaster Monitoring and Alerts). The goal of this call is to propose new tools and methods to help CEMADEN to improve the preparedness for natural disasters in Brazil. In the context of this project (The project webpage is at http://disaster-robotics-proalertas.github.io/), the authors of this survey have developed some robotic tools to simplify the information gathering for disaster prevention and to be used for disaster mitigation and response. Two main robotic platforms are being used in the project: USVs for flooding prevention and response, and UAVs for mapping areas prone to landslides. However, description of the later is out of the scope of this paper.

This section presents the current and near future contributions of the authors in the field of USVs, with focus to disaster applications. These contributions are organized as: boat prototypes (Section 7.1), new payload (Section 7.2), realistic robotic simulation for flooding scenarios (Section 7.3), and USV software (Section 7.4).

### 7.1. Boat Prototypes

*N-Boat II—The Sailboat Robot*: This USV is currently under development by Natalnet partner Labs. The N-Boat II is a reusable and self-sustaining USV [247]. It is a 2.5 m long vessel (0.8 m width) and weighs about 150 kg—including the hull (app. 65 kg), two 104 A/H nautical batteries (50 kg), and the keel/blade (35 kg). It has been developed since 2012, following the lessons learned from the N-boat I USV [248]. It can withstand open seas and strong winds and support long-term missions autonomously [249]. Its main applications will focus on monitoring tasks, which include environmental protection and border surveillance, as well as natural disaster mitigation through long-term extreme weather forecasting.

*Platypus USV Platforms*: The project also contains two Lutra Airboats and one Lutra Prop, with a differential drive system, from Platypus Limited Liability Company (LLC) (http://senseplatypus.com/). Lutra boats are approximately 1.5 m long, with about 8kg of weight, supporting a payload of about 1.5 kg. We have made modifications for both software and payload of the boats. For instance, we have adapted the Ardupilot autopilot boards to perform basic waypoint navigation, return to launch, and maneuvers compensation by software, integrated with Robot Operating System (ROS), a robotics middleware. Therefore, we intend to soon perform different path planning and obstacle avoidance according to COLREGS. We have also improved the communication capabilities between the boats: in addition to the Wi-Fi connectivity, we integrated long-range radios for basic telemetry and inter-boat communication.

### 7.2. Fleet Modifications

We have proposed a new payload system for the N-Boat II [250], including environmental monitoring sensors and real-time and online communications. The new N-Boat II sailboat architecture is vital for autonomous long-term missions since it can stream available sensor data over the Internet to DM and environmental monitoring agencies. The idea is to prepare the N-Boat II to be used as an early warning system for environmental and natural disasters.

For the Lutra boats, we integrated an embedded image processing payload, consisting of a sealed acrylic box with a Zed^®^ RGBD camera (RGB + depth) (http://www.stereolabs.com), capable of outdoor use with a depth range of about 20m. For image processing, we are using NVIDIA^®^’s Jetson TX2 board with Pascal GPU architecture, including 256 CUDA cores. This payload is designed to run Convolutional Neural Networks (CNN), in applications for real-time obstacle detection and other image processing tasks. Finally, the boats can also carry bathymetry sensors and a system to collect water samples remotely. In the future, we intend to include an ADCP sensor to measure water flow—an important parameter for hydrologic studies associated with extreme weather.

### 7.3. Simulation of Scenarios Involving USVs and Floods

During a flood, the USV might encounter strong water current, winds, water vortexes, and floating debris that might jeopardize the mission. Before testing with real boats, the software designers must have tools to test the USVs safely, in simulation scenarios. Simulators intend to prepare the USV and the rescue team for the actual mission. Presently, there are few open source robotics simulators and fewer which simulate aquatic environments with enough accuracy to simulate a flooding disaster. One of our research goals is to design a robotic simulator to support accurate wind, wave, and hydrologic models to mimic a flooding scenario [251].

Fluid simulation models such as HEC-RAS (http://www.hec.usace.army.mil/software/hec-ras/) and Openfoam (https://openfoam.org/) were incorporated into the Gazebo (http://gazebosim.org/) robotic simulator. Also, several different USV models (e.g., airboat, differential boat, rudder boat, and sailboat) were designed to test the performance of those models under the same simulated disaster scenario. Some of these USV models are being calibrated with the actual boats described in Section 7.1, through several field trials to generate more accurate USV models. Therefore, our simulation environment allows not only for testing different boats but also to test a series of algorithms such as control strategies, path planning, and obstacle avoidance under controlled and repeatable disaster environments. Soon, we intend to benchmark different control and obstacle avoidance strategies for boats in flooding environments using this simulator.

### 7.4. Proposed Control, Computer Vision, and Planning Applications

For the N-Boat II, the idea is to design and develop a control system capable of controlling the sailboat displacement with enough generality, in such a way that it can be implemented in other sailboats [249]. Another goal is to be able to navigate using low-cost sensors such as a compass, GPS, and windsock. We have tested three types of controllers using fuzzy, Proportional-Integral-Derivative (PID), and an empirically defined simple proportional control. We have developed a mathematical model for the system architecture and control paradigms. The main advantage of having such a model established is that we could create and validate a simulation environment for the sailboat, accelerating the design of the currently used control laws, avoiding unnecessary field trials with the sailboat and reducing logistics costs. The PID controller is currently implemented in the N-Boat II prototype and working robustly.

For Lutra boats, a vision-based system using an RGBD camera for real-time obstacle detection is under development. An obstacle avoidance system is essential because, in its current autonomy level, the USV features a waypoint navigation strategy. The problem is that the autopilot system navigates in a straight line between waypoints, assuming no obstacles in the path. In a disaster scenario such as flooding, we cannot assume this is true because there are usually moving debris, carried by the strong water flow. As CNN are state of the art for object detection and recognition [252,253,254], our system uses an NVIDIA^®^ Jetson board for CNN processing and obstacle detection. Once an obstacle is detected, it can be classified either as debris, survivors, or other vessels. One mandatory requirement is that the obstacle avoidance procedure must follow the COLREGS and maintain a safe distance from eventual survivors—to avoid injuring them. The integration of obstacle detection, avoidance, and COLREGS-based path planning is currently under development.

## 8. Conclusions

In this paper, we performed a review of state of the art in USVs for DM, focusing on both natural or man-made disasters. Most USV reviews focus on GNC, while the research involving USVs for DM is spread across different DM oriented publications, with focus on more than one unmanned vehicle and varying degrees of depth. This review is the first focused specifically on USVs for DM.

As the present research features a broad research problem, its inherent challenge is the capacity to encompass all works on the field. We did our best to cover the field, but we may not have covered all of it. We might have missed works as the project evolved and tasks were subdivided. Another challenge while studying USVs is the naming convention, which is not uniform—e.g., unmanned marine/surface crafts, vessels, vehicles, or autonomous or unmanned boats. Such a lack of naming conventions for USVs leads to a series of research problems, including exceeding character limits in search fields and difficulty while narrowing down relevant works. Furthermore, search engines can associate such acronyms with studies in physics, medicine, economics, history, and other unrelated fields. One way to address this is to use exclusion keywords in the search—e.g., excluding the word “blood” from the search. The term marine vehicle is also ambiguous, which may lead to UUVs or USVs. Similar problems occur with disaster keywords such as “flood”, which is associated with network security. The use of exclusion words here also applies—e.g., excluding DDoS, hacking. Still, the primary research goal was to put USVs for DM in the spotlight—away from the generic UMV nomenclature. In this sense, we believe to have succeeded.

This paper presents a list of current DM applications for USVs. While there are plenty of promising works on the field globally, most of them are still experimental and not fully developed. Among the uses of USVs in the DM process, we highlight some of the mature applications available—e.g., SAR; extreme weather forecast; seismic event forecast; structural inspection; and disaster impact assessments on the environment. Other recurrent motivations to use USVs are to move through dangerous scenarios where manned surface vehicles cannot go such as hurricanes or extreme weather regions at sea, to find routes through debris, or to perform inspection nearby dams or bridges. Also, in the event of an environmental disaster, such as large oil spills, USVs can quickly move toward the accident zone and perform environmental damage assessments such as measuring water contamination and water-life impact assessments. One of the surprising discoveries involves floods. Even though they are a recurrent motivator for USV research, the subject is not directly addressed. Often, the USV research addresses floods only in the recovery phase.

In general, most researchers focus on technology and not on disaster-oriented mission results, even though disaster scenarios recurrently motivate them. Consequently, few papers test USVs in disaster sites or similar conditions. Therefore, it means that most papers do not address the effectiveness or efficiency of the USV in such extreme conditions. We speculate that as the research focus is often technology-oriented—i.e., typical control, GNC, multi-robot cooperation, and other fundamental research problems—tests in disaster scenarios are left as secondary future work. Another possible explanation is that research teams are usually not multidisciplinary enough to address the multifaceted research challenges involving DM. Even if a research team plans to do that, there are many problems to such a challenging endeavor. The main one is the cost of reproducing such extreme conditions in actual field trials. Today there are numerous competitions involving heterogeneous robotic systems which include USVs and few testing sites which emulate disaster scenarios. However, the cost of transporting the team and the robots to such sites is often unfeasible for many research institutions. If a solution is not found, the unavoidable consequence of such a significant limitation will be the lack of reliability of USVs in harsh DM scenarios.

The present work identified a trend in DM involving aquatic environments: the use of heterogeneous fleets of unmanned systems working together, with promising results and applications. USVs, UAVs, and UUVs have complementary advantages and weakness. Even though UUVs and UAVs have their limitations, they can provide different perspectives to disaster sites by performing measurements and going to regions where USVs cannot go. USVs can carry a large payload, depending on its size, and provide energy and communications infrastructure for UUVs and UAVs as a moving station. However, the use of USVs for such purpose is still evolving, and live tests are still restricted to competitions. Another problem constantly occupying researchers is conformance with international naval regulations. Among them, the COLREGS and country-specific rules which must be respected to avoid collision accidents. USVs share similar concerns with autonomous cars, where the discussion of responsibility in case of accidents is still not clarified. Thus, regulatory and legal concerns bring together a considerable amount of problems which are being individually addressed, case by case, by each country and research group.

## Figures and Tables

**Table 1:** List of keywords used in research databases.

USV Related Terms	Disaster-Related Terms
unmanned vessel, unmanned boat, autonomous vessel, autonomous boat, autonomous craft, unmanned craft, Unmanned Surface Vehicles (USVs), Autonomous Surface Vehicles (ASVs), Unmanned Marine Vehicles (UMVs), Unmanned Surface Crafts (USCs), Autonomous Surface Crafts (ASCs), Unmanned Surface Vessel, Autonomous Surface Vessel, Underactuated surface vessel, micro unmanned surface vessels	Disaster Prevention, Disaster Recovery, Disaster Relief, disaster response, emergency response, disaster prevention, disaster, inspection, reconnaissance & mapping, monitoring, surveillance, Search And Rescue (SAR), hurricane, tsunami, earthquake, floods, extreme event, emergency response

**Table 2:** Characteristics of work related to natural disasters grouped by task, where “T. & E.” stands for “Tsunami & Earthquake”, “L & E” stands for “Landslides & Erosion”, and “NR” stands for “Not Reported”.

Task	Reference	Year	USV Name	USV Type	Test Location
T. & E.	[53,54]	2012	*NR*	Buoy	Tonakai segment, Nankai Trough, Japan
	[55]	2013	*NR*	Monohull vessel	Japan Trench
	[56]	2015	MERMAID	Argo floats	Mediterranean Sea
	[57,58]	2016	*NR*	Wave Glider	*NR*
	[59]	2014	*NR*	Wave Glider	Gulf of Mexico and US eastern seaboard
	[60]	2016	SV2	Wave Glider	Loch Ness, Scotland
Hurricanes	[61]	2008	AEOS-1	Twin-hull catamaran	Marco Island, USA
	[62,63,64]	2009	Sea-RAI	Twin-hull catamaran	Galveston, Texas, USA
	[65,66]	2012	EMILY	Monohull vessel	Simulator
	[67]	2014	Benjamin	Wave Glider	New Caledonia
	[68]	2015	SV2	Wave Glider	Caribbean Sea
	[69]	2016	*NR*	Wave Glider	Okinawa, Japan
Floods	[70,71]	2012	*NR*	Airboat	Philippines and New York, USA
	[8,72]	2015	*NR*	Monohull vessel	Province of Ancona, Italy
	[73,74]	2016	*NR*	Monohull vessel	*NR*
	[75]	2018	*NR*	Monohull vessel	Port Fourchon, Louisiana, USA
L. & E.	[76]	2009	ROAZ II	Twin-hull catamaran	Portuguese Tua River
	[77]	2008	ROAZ II	Twin-hull catamaran	Douro estuary sand spit and
					Vila Nova de Gaia coast, Portugal

**Table 3:** Characteristics of work related to chemical hazards grouped by task, where “NR” stands for “Not Reported”.

Task	Reference	Year	USV Name	USV Type	Test Location
Detection	[102,103]	2011	HydroNet	Twin-hull catamaran	Livorno, Italy
	[104]	2012	ASV-Victoria	Twin-hull catamaran	Louisiana, USA
	[105]	2016	*NR*	*NR*	*NR*
	[106]	2017	HydroNet	Twin-hull catamaran	Livorno, Italy
Monitoring	[94]	2013	*NR*	Wave glider	Gulf of Mexico
	[107]	2015	*NR*	Buoy	Biograd na Moru, Croatia
	[101]	2016	BUSCAMOS-Oil	Monohull vessel	Cartagena, Spain
	[108]	2016	C-Enduro/C-Worker	Twin-hull/Monohull	Gulf of Mexico
	[93]	2017	PlaDyPos	Buoy	Cartagena, Spain
Tracking	[109,110]	2012	SOTAB-II	Buoys	*NR*
	[111,112]	2012	SOTAB-II	Buoys	Osaka Bay, Japan
	[113,114]	2015	SOTAB-II	Yacht-shaped	Kobe, Japan
	[115]	2015	*NR*	Twin-hull catamaran	Simulator
	[116]	2017	*NR*	Monohull vessel	Simulator
	[117]	2017	*NR*	Twin-hull catamaran	Oahu, Hawaii
Caging	[118,119]	2010	USC RESL	Monohull vessel	Los Angeles, USA
	[120]	2011	USC RESL	Monohull vessel	Los Angeles, USA
	[121]	2011	*NR*	Monohull vessel	*NR*
	[95]	2012	*NR*	Containment boom	*NR*
Removing	[122]	2007	EU-MOP	Monocat and catamaran	*NR*
	[123]	2008	EU-MOP	Monocat and catamaran	*NR*
	[124,125]	2015	*NR*	Scaled Zodiac	Madrid, Spain
	[126]	2015	PUS	Monohull vessel	*NR*
	[127]	2016	HaiTeng 01	Monohull vessel	Shenzhen Bay, China

**Table 4:** Characteristics of work related to chemical hazards grouped by task, where “NR” stands for “Not Reported”.

Reference	Year	USV Name	USV Type	Test Location
[146,147]	2006	OASIS	Platform	Chincoteague Bay, USA
[148,149]	2007	OASIS	Platform	Chesapeake Bay, USA
[150]	2007	Roboduck-II	Monohull vessel	Redondo Beach, USA
[151]	2008	MARTA/OASIS	Platform	Chesapeake Bay, USA
[152]	2009	MARTA/OASIS/RSB	Platform/Kayak	Chesapeake Bay, USA
[153,154,155,156]	2009	Lake Wivenhoe ASV	Twin-hull catamaran	Lake Wivenhoe, Australia
[157,158]	2012	Lizhbeth	Twin-hull catamaran	Lake Zürich
[159]	2014	*NR*	Twin-hull catamaran	Simulation
[160]	2017	*NR*	*NR*	Ypacarai Lake, Paraguay (simulation)
[161]	2016	Grace	Gliding robotic fish	Wintergreen Lake, Michigan
[162]	2016	Wave glider	Solar wave glider	*NR*

**Table 5:** USVs Capabilities for Disaster Robotics.

Operation	DM Phase	Maturity
Storm and Hurricane Forecast	Preparedness, Mitigation & Response [61,62,63,64,65,66,67,68,69]	mature
Structural Inspection	Preparedness, Mitigation & Response [19,61,62,63,64,79,83,175,176,177,178,179,180,181,182,183,184]	mature
Search & Rescue	Response [21,66,189,190,191,192,193,194,197,198,199,200,201,202,203,204,207,209,210,211,212,213,214,216,217,218,219,220,221,222,223,224,226,227,228]	mature
Assess Water-Life Impacts after Disaster	Recovery [93,94,101,107,108]	mature
Online Detection of Water Contaminants	All [102,103,104,105,106]	mature
Tsunami Forecast	Preparedness, Mitigation & Response [53,54,55,56,57,58,59,60]	deployed
Landslide and Erosion Detection	Preparedness & Mitigation [76,77,91]	deployed
Detection & Monitoring HAB	Preparedness, Mitigation & Response [146,147,148,149,150,151,152,153,154,155,156,157,158,159,160,161,162]	deployed
Flood Risk Monitoring	Preparedness, Mitigation & Response [8,70,71,72,73,74,75]	deployed
Tracking & Containment of Water Plumes	All [95,109,110,111,112,113,114,115,116,117,118,119,120,121]	experimental
Radioactivity Detection	All [166,167,168,170,171]	experimental

## Abbreviations

The following abbreviations are used in this manuscript:

3D	Three Dimensional
COLREGS	COLlision REGulations at Sea
CRASAR	Center for Robotic-Assisted Search and Rescue
DM	Disaster Management
FP7	Seventh Framework Program
GNC	Guidance Navigation and Control
GPS	Global Positioning System
HAB	Harmful Algal Bloom
ICARUS	Integrated Components for Assisted Rescue and Unmanned Search
LiDAR	Light Detection And Ranging
OASIS	Ocean-Atmosphere Sensor Integration System
SAR	Search and Rescue
UAV	Unmanned Aerial Vehicle
UMV	Unmanned Marine Vehicle
USV	Unmanned Surface Vehicle
UUV	Unmanned Underwater Vehicle
